# A transcriptomic pan-cancer signature for survival prognostication and prediction of immunotherapy response based on endothelial senescence

**DOI:** 10.1186/s12929-023-00915-5

**Published:** 2023-03-28

**Authors:** Zhengquan Wu, Bernd Uhl, Olivier Gires, Christoph A. Reichel

**Affiliations:** 1 Department of Otorhinolaryngology, Ludwigs-Maximilians-University Medical Centre, Marchioninistr. 15, 81377 Munich, Germany; 2 Walter Brendel Centre of Experimental Medicine, University Hospital, LMU Munich, Munich, Germany; 3 German Cancer Consortium (DKTK), Partner Site Munich and German Cancer Research Center (DKFZ), Heidelberg, Germany

**Keywords:** Endothelial cell senescence, Pan-cancer analysis, Immunotherapy, Prognosis, scRNA-seq

## Abstract

**Background:**

The microvascular endothelium inherently controls nutrient delivery, oxygen supply, and immune surveillance of malignant tumors, thus representing both biological prerequisite and therapeutic vulnerability in cancer. Recently, cellular senescence emerged as a fundamental characteristic of solid malignancies. In particular, tumor endothelial cells have been reported to acquire a senescence-associated secretory phenotype, which is characterized by a pro-inflammatory transcriptional program, eventually promoting tumor growth and formation of distant metastases. We therefore hypothesize that senescence of tumor endothelial cells (TEC) represents a promising target for survival prognostication and prediction of immunotherapy efficacy in precision oncology.

**Methods:**

Published single-cell RNA sequencing datasets of different cancer entities were analyzed for cell-specific senescence, before generating a pan-cancer endothelial senescence-related transcriptomic signature termed *EC.SENESCENCE.SIG*. Utilizing this signature, machine learning algorithms were employed to construct survival prognostication and immunotherapy response prediction models. Machine learning-based feature selection algorithms were applied to select key genes as prognostic biomarkers.

**Results:**

Our analyses in published transcriptomic datasets indicate that in a variety of cancers, endothelial cells exhibit the highest cellular senescence as compared to tumor cells or other cells in the vascular compartment of malignant tumors. Based on these findings, we developed a TEC-associated, senescence-related transcriptomic signature (*EC.SENESCENCE.SIG)* that positively correlates with pro-tumorigenic signaling, tumor-promoting dysbalance of immune cell responses, and impaired patient survival across multiple cancer entities. Combining clinical patient data with a risk score computed from *EC.SENESCENCE.SIG,* a nomogram model was constructed that enhanced the accuracy of clinical survival prognostication. Towards clinical application, we identified three genes as pan-cancer biomarkers for survival probability estimation. As therapeutic perspective, a machine learning model constructed on *EC.SENESCENCE.SIG* provided superior pan-cancer prediction for immunotherapy response than previously published transcriptomic models.

**Conclusions:**

We here established a pan-cancer transcriptomic signature for survival prognostication and prediction of immunotherapy response based on endothelial senescence.

**Supplementary Information:**

The online version contains supplementary material available at 10.1186/s12929-023-00915-5.

## Introduction

Cellular senescence has recently been included in the ‘hallmarks of cancer’ [1, 2] as a fundamental characteristic of malignant tumors [3]. This term describes an irreversible state of cell cycle arrest, which is thought to be evolved as a protective biological mechanism facilitating the clearance of diseased or dysfunctional cells. Cellular senescence commonly arises in ageing organisms but can also be induced by more specific conditions such as lack of nutrient supply or direct cell damage. As a result, senescent cells typically show alterations in cell morphology or metabolism and demonstrate a ‘senescence-associated secretory phenotype’ (SASP), which is associated with the release of a broad range of bioactive substances into the extracellular space [4, 5]. Whereas cellular senescence in cancer has initially been regarded as beneficial [6], there is accumulating evidence that this cellular state elicits pro-tumorigenic effects including stimulation of tumor cell proliferation, invasion, and metastasis, promotion of tumor angiogenesis, as well as interference with tumor immunity [7–9]. Importantly, tumor cells are able to adopt transitory and reversible senescent states that critically contribute to therapy resistance [10, 11].

The microvascular endothelium regulates nutrition and oxygen supply to cancer cells and controls immune surveillance, thus representing an integral component of the tumor environment. In contrast to healthy tissues, however, the tumor microvasculature is disorganized and consists of immature vessels with disrupted endothelial junctions, incomplete basement membrane coverage, and lacking pericyte sheath. Consequently, vessels of growing tumors are progressively low perfused and highly permeable, which causes tumor hypoxia, supports cancer cell dissemination, and promotes inadequate immune responses. From a therapeutical perspective, this favors resistance to different modalities of cancer treatment including radio- and cytostatic therapy, and, in particular, immunotherapy [12]. In this context, it is interesting that besides cancer cells, other cell populations in the tumor environment including microvascular endothelial cells undergo senescence [13–15]—a process potentially linked to the cancer hallmarks ‘inducing angiogenesis’, ‘activating invasion and metastases’, and ‘tumor-promoting inflammation’.

Individual survival prognostication and prediction of response to therapy are critical for the development of personalized treatment concepts in precision oncology. With respect to the fundamental role of the microvascular endothelium in cancer (immuno)biology and treatment resistance, we hypothesize that senescence of tumor endothelial cells (TEC) substantially participates in tumor progression and immunotherapy efficacy in solid cancers, hence serving as a promising target for survival prognostication and immunotherapy response prediction.

## Methods

### Pan‑cancer scRNAseq datasets and processing

To develop a TEC-specific senescence-related transcriptomic signature (*EC.SENESCENCE.SIG*), we collected 18 scRNAseq datasets containing tumor, stromal, and immune cell data. These 18 scRNAseq datasets included 15 types of cancer, including ovarian cancer (OV), pancreatic cancer (PAAD), prostate cancer (PRAD), melanoma (SKCM), stomach cancer (STAD), ocular melanomas (UVM), basal-cell carcinoma (BCC), bladder cancer (BLCA), breast cancer (BRCA), colorectal cancer (CRC), head and neck cancer (HNSC), kidney clear cell carcinoma (KIRC), lower grade glioma (LGG), liver cancer (LIHC), and lung adenocarcinoma (LUAD). Raw data were downloaded from Gene Expression Omnibus (GEO, https://www.ncbi.nlm.nih.gov/geo/), The European Genome-phenome Archive (EGA, https://ega-archive.org/), and Array Express (https://www.ebi.ac.uk/arrayexpress/). ScRNAseq data processing was performed using the R ‘Seurat’ package (https://satijalab.org/seurat/) as described in the package tutorial. In brief, cells with gene expression < 300 genes or > 6500 genes and mitochondrial gene expression > 10% were excluded, hence including the vast majority of cells in the employed datasets. We further applied the SCTransform function (https://satijalab.org/seurat/articles/sctransform_vignette.html) to normalize and scale raw counts followed by principal component analysis (PCA). The R ‘Harmony’ package (https://portals.broadinstitute.org/harmony/articles/quickstart.html) was used to remove batch effects across dissociated scRNAseq raw data if required. Employing unsupervised cluster analysis and unified manifold approximation and projection (UMAP), we identified discrete cell clusters in each of the scRNAseq datasets. We then annotated each cell cluster based on known cell type marker genes or annotation that come with the downloaded datasets. Furthermore, the differential genes of each cell type were identified using the ‘FindAllMarkers’ function in R ‘Seurat’ package and only genes that were enriched and expressed in at least 25% of the cells of at least one cell type and with a log fold change higher than 0.25 were retained (default values of this package).

### Pathway activity and cell–cell communication analysis

For pathway activity analysis, normalized gene expression data from each cell cluster (scRNAseq) or patients (bulk-seq) were implemented in R ‘GSVA’ package (https://github.com/rcastelo/GSVA) to evaluate the enrichment of related gene sets. The gene sets used for Gene Set Variation Analysis (GSVA) were downloaded from the Molecular Signatures Database (MSigDB) website (https://www.gsea-msigdb.org/gsea/msigdb). Endothelial cells in scRNAseq datasets were divided into high- and low-senescent tumor endothelial cells (HS-TEC and LS-TEC, respectively) according to the GSVA score of a senescence-related transcriptional signature termed ‘*FRIDMAN. SENESCENCE.UP*’ [16]. Cellchat (Version 1.5.0, https://github.com/sqjin/CellChat) was applied to analyze cell–cell communication using the normalized gene expression matrix. Interaction pairs between HS-TEC and other cell types with P-values less than 0.01 were retained.

### Pan-cancer bulk-seq datasets and immunotherapy treated cohort

The Cancer Genome Atlas (TCGA) transcriptome data for all cancer types were downloaded from UCSC XENA website (https://xenabrowser.net/datapages/) and only patients with available overall survival data were selected for further data analysis. The TCGA pan-cancer total mutation burden (TMB) data was obtained from the cBioPortal for Cancer Genomics (cBioPortal, https://www.cbioportal.org). In addition, we also collected the clinical and transcriptomic data of CGGA (Chinese Glioma Genome Atlas, n = 651, downloaded from CGGA website (http://www.cgga.org.cn/). The transcriptomic data and clinical features of METABRIC (Molecular Taxonomy of Breast Cancer International Consortium, n = 1868) and prad-su2c-2019 (prostate cancer, n = 444) were downloaded from cBioPortal (https://www.cbioportal.org). Furthermore, clinical and transcriptome data of other cancer types were downloaded from Gene Expression Omnibus (GEO), including GSE13507 (bladder cancer, n = 165), GSE17538 (colorectal cancer, n = 238), GSE19423 (bladder cancer, n = 48), GSE30219 (lung cancer, n = 278), GSE72094 (lung cancer, n = 398), GSE138866 (ovarian Cancer, n = 130). For anti-programmed death-1 (PD-1)/programmed cell death ligand 1 (PD-L1) or anti-cytotoxic T-lymphocyte-associated Protein-4 (CTLA-4) immunotherapy-treated cohorts, we systematically retrieved and collected gene expression data and prognostic information of samples from 13 anti-PD-1/PD-L1- or anti-CTLA-4-treated cohorts, and only treatment-naïve patients were retained for further analysis, including 7 melanoma cohorts (Hugo SKCM, Liu SKCM, Gide SKCM, Riaz SKCM, Van SKCM, PUCH SKCM, Auslander SKCM), 2 urothelial carcinoma cohorts (Mariathasan UC, Snyder UC), 1 glioma cohort (Zhao GBM), 1 gastric cancer cohort (Kim GC), 1 lung cancer cohort (Jung NSCLC), and 1 renal cell carcinoma cohort (Bruan RCC). All related processed data of immunotherapy cohorts were downloaded from GEO or online correspondingly published article (Additional file 1: Table S3).

### Construction of machine learning model for predicting response of immune checkpoint blockade

To investigate the predictive power of *EC.SENESCENCE.SIG* for response to anti-PD-L1/PD-1 or anti-CTLA-4 immune checkpoint blockade, we systemically collected 13 suitable cohorts with transcriptome sequencing data and clinical results of immunotherapy. First, seven immunotherapy datasets were combined into a merged dataset (n = 775), including Hugo SKCM (n = 26), Liu SKCM (n = 121), Gide SKCM (n = 73), Riaz SKCM (n = 49), Van SKCM (n = 36), Mariathasan UC (n = 298), and Bruan RCC (n = 172). The ‘ComBat’ algorithm in the R ‘sva’ package (https://bioconductor.org/packages/release/bioc/html/sva.html) was used for removing batch effects. Subsequently, the merged dataset was randomly split into training (n = 620, 80%) and validation sets (n = 155, 20%). The endothelial cell senescence related gene signature was applied to construct a prediction model in the training set using ten machine learning algorithms, including support vector machine (SVM), Naïve Bayes (NB), random forest (RF), k-nearest neighbors (KKNN), AdaBoost Classification Trees (AdaBoost), boosted logistic regressions (LogiBoost), Gradient Boosting Machines (GBM), Bagged CART, Nearest Shrunken Centroids (PAM), and Neural Network. All these ten classification algorithms were implemented using the R ‘Caret’ package (https://topepo.github.io/caret/) and each algorithm was validated using five times repeated tenfold cross validation. In order to improve the accuracy of the models, we repeated the optimization process five times with different random seeds. Subsequently, these ten models were implemented into validation sets to compare their performance. The model with the highest accuracy was selected as a final classification model. Further, the predictive ability of the final model was evaluated using six independent testing sets, including PUCH SKCM (n = 49), Auslander SKCM (n = 14), Snyder UC (n = 25), Zhao GBM (n = 17), Kim GC (n = 45), and Jung NSCLC (n = 27). To evaluate the predictive accuracy of our final model, we compared our model with six other published pan-cancer predictive models to evaluate the response to immunotherapy in testing sets. All the codes and algorithms for the above six models were derived from relevant published articles.

### Functional analysis of endothelial cell senescence related signature (*EC.SENESCENCE.SIG*)

*EC.SENESCENCE.SIG* was functionally annotated by assessing the enrichment of Gene Ontology (GO) terms (biological process, cellular component terms, molecular functions) and Reactome pathways. The R ‘clusterProfiler’ package was used to visualize the related GO terms or Reactome pathways with adjusted P value less than 0.05.

### Pan-cancer analysis of Gene Set Enrichment Analysis (GSEA) and immune infiltration quantification

First, the GSVA *EC.SENESCENCE.SIG* score of every patient of the TCGA pan-cancer cohorts was calculated using R ‘GSVA’ package. The patients of each cancer type were further divided into two groups based on the median of GSVA *EC.SENESCENCE.SIG* score. Subsequently, we used GSEA to analyze the enrichment of differentially expressed genes between two groups in selected tumor-promoting pathways that were downloaded from the msigDB website. For the quantification of immune cell infiltration, the Cell-type Identification by Estimating Relative Subsets of RNA Transcripts (CIBERSORT) algorithm [17] and the LM22 signature matrix were applied to estimate immune infiltration according to the transcriptome data (TPM value) of TCGA pan-cancer cohorts.

### Construction and validation of the TEC-related prognostic model

Among the 102 genes in *EC.SENESCENCE.SIG*, the Least Absolute Shrinkage and Selection Operator (LASSO) regularized regression with tenfold cross-validation was performed to select smaller features that are most associated with overall survival of patients in TCGA pan-cancer cohorts. We conducted the LASSO regularized regression using the R ‘glmnet’ package and 50 genes were screened out and inputted into further analysis. We therefore used the gene expression of these 50 genes to construct a multivariate Cox proportional hazard regression model with a stepwise method (combination of the forward and backward selection) for overall survival of TCGA pan-cancer training sets (n = 6746, 80%). Based on the multivariate Cox proportional hazard regression model, the *EC.SENESCENCE.SIG*-related risk score of each patient was calculated by the formula as follow: *EC.SENESCENCE.SIG*-related risk score (EC sene score) = Σ (cox regression coefficient) × (normalized expression level of each gene). We obtained the risk score of every patient in the TCGA pan-cancer training sets, TCGA pan-cancer testing sets, and external validation sets using the above formula. Patients were dichotomized into a high-risk group and a low-risk group using the median risk score and subsequently analyzed for the difference of overall survival using R ‘survival’ package. Furthermore, the area under the curve (AUC) was calculated to evaluate the sensitivity and specificity of the above prognostic model using R ‘timeRoc’’ package or R ‘pROC’ package. To visualize the related prognostic model, a nomogram and a forest plot were graphed using R ‘rms’ package and R ‘forestplot’ package. For feature selection, we used R package ‘randomForestSRC’ (random forest), R package ‘XGBoost’, and R ‘glmnet’ package to quantify the importance of each gene in *EC.SENESCENCE.SIG* for patients’s overall survival in TCGA pan-cancer cohorts.

### Meta-analysis

To elucidate the combined prognostic value of the modified nomogram model in the ten different datasets, a prognostic meta-analysis was conducted using R ‘meta’ package. Subsequently, the pooled hazard ratio (HR) values were calculated using random effects and synergistic effects models.

### Statistical analysis

All statistical analyses were performed using R v4.2.1 (https://www.r-project.org). Differences in survival between the two groups were assessed using Kaplan–Meier curves and the log-rank test. Univariate and multivariate Cox regression analyses were used to determine prognostic factors. For correlation analysis, correlation coefficients were calculated using Pearson for normally distributed data and Spearman for non-normally distributed data. For analysis of differences between two groups of data, unpaired Student’s t-test and Mann–Whitney U-test were used for normally and non-normally distributed variables, respectively. To compare more than two groups, one-way analysis of variance (ANOVA) and Kruskal–Wallis’s tests were used as parametric and nonparametric methods, respectively. The adjusted p-value (FDR) was calculated by the Benjamini–Hochberg correction method. The error bars represent the standard deviation of the mean. P < 0.05 was considered statistically significant unless mentioned otherwise.

## Results

### Senescence status of individual cell populations in the tumor environment

**Fig. 1:**
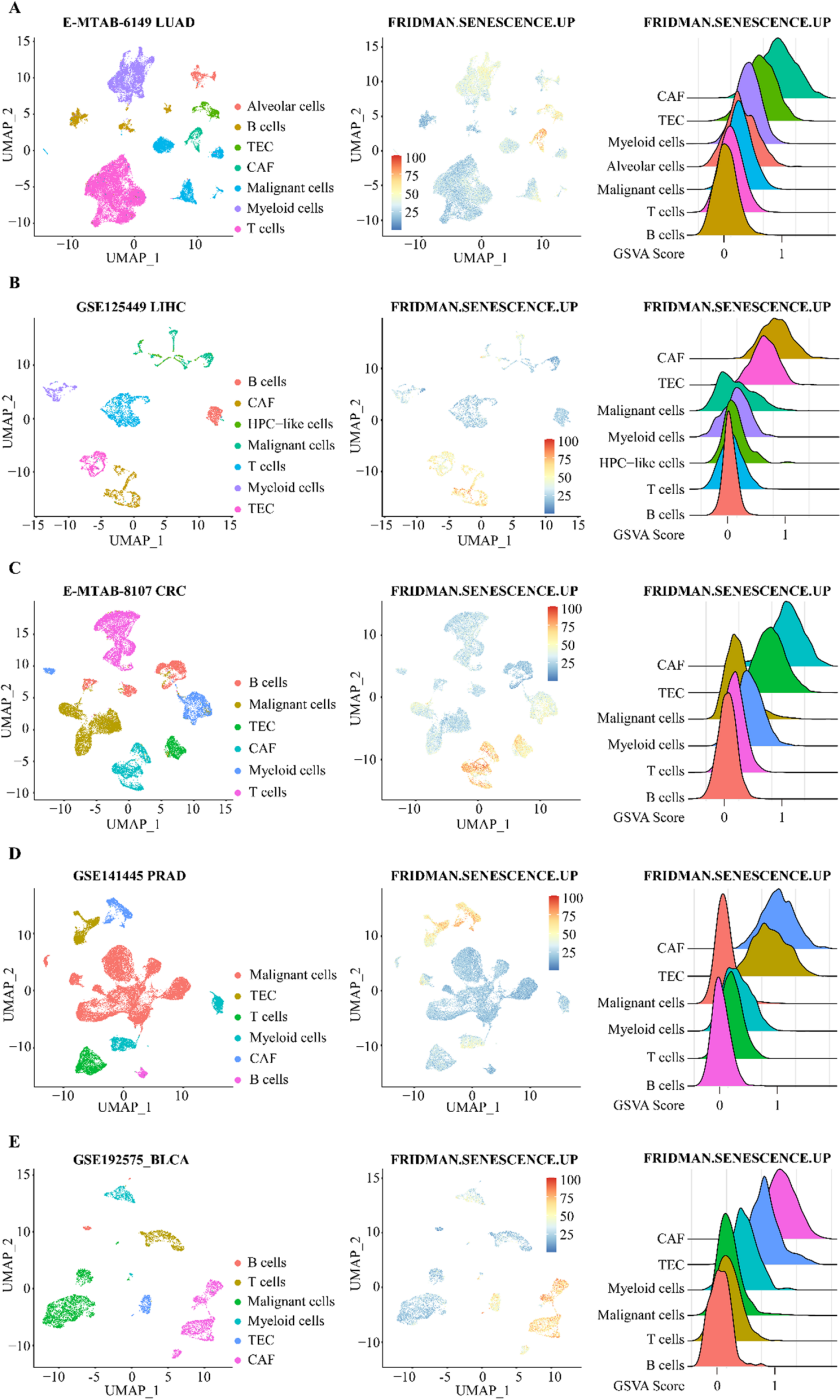
Evaluating the senescence status of individual cell types in the tumor environment. Unified manifold approximation and projection (UMAP) plots show integrated datasets of clusters and cluster cell type annotations from 5 scRNAseq datasets of different tumor entities (**A**–**E**, left). Feature plots show the enrichment of senescence-related gene sets per cell in pseudocolors (**A**–**E**, middle). Ridge plots (**A**–**E**, right) show the distribution of the senescence score across cell clusters; *CAF* cancer-associated fibroblast, *TEC* tumor endothelial cells, *HPC* hepatic progenitor cells

In the present study, we hypothesize that senescence-related transcriptomic changes in TEC represent a potential target for survival prognostication and prediction of immunotherapy response in cancer. To prove this hypothesis, we implemented five published single-cell ribonucleic acid sequencing (scRNAseq) datasets of different cancer entities including lung cancer, liver cancer, colorectal cancer, prostate cancer, and bladder cancer. As a measure of cellular senescence, we used an established senescence-related gene set [16] (*FRIDMAN.SENESCENCE.UP*). In a first approach, we extracted and clustered high-quality cells of all datasets, as presented by unified manifold approximation and projection (UMAP) plots (Fig. 1a–e). Subsequent gene-set variation analyses (GSVA) in these cells based on *FRIDMAN.SENESCENCE.UP* revealed that TEC exhibit higher GSVA scores than tumor cells or other cell populations in the tumor environment including myeloid cells, T cell, and B cells, except cancer-associated fibroblasts (CAF; Fig. 1a–e). Thus, TEC exhibit the highest cellular senescence levels in the vascular compartment of malignant tumors.

Senescent cells are characterized by a phenotype that is associated with the secretion of bioactive substances capable of modulating the activation status of different cells [5]. To further explore the overall communication between senescent TEC and other cell populations in the tumor environment, we divided TEC into high-senescent (HS-TEC) and low-senescent cells (LS-TEC) based on the median of GSVA scores for *FRIDMAN.SENESCENCE.UP* (Additional file 1: Table S1) in three scRNAseq datasets (E-MTAB-6149, lung cancer), (GSE125449, liver cancer), and (GSE195832, head and neck cancer) containing large numbers of endothelial cells. As a measure of cell–cell interactions, we then evaluated communication networks of HS-TEC by network analysis and pattern recognition approaches [18]. Here, we demonstrate that HS-TEC establish a higher number of interactions in the tumor microenvironment as compared to LS-TECs (Additional file 1: Fig. S1a–c), predominantly communicating with immune cells (Additional file 1: Fig. S1a–c). For this purpose, HS-TEC release the cytokine *macrophage migration inhibitory factor* (MIF) and β-galactosid-binding lectins (galectin; Additional file 1: Fig. S2a–c), utilizing five pathways that include MIF-CD74/C-X-C motif chemokine receptor 4 (MIF-CD74^+^CXCR4), MIF-CD74/CD44 (MIF-CD74^+^CD44), Galectin9-TIM3 (LGALS9-HAVCR2), Galectin9-CD45 (LGALS9-CD45), and Galectin9-CD44 (LGALS9-CD44; Additional file 1: Fig. S2a–c). Hence, our data suggest that senescence of TEC particularly facilitates interactions with immune cells by involving MIF- and galectin-dependent pathways.

### Development of a pan-cancer transcriptomic signature based on endothelial senescence

**Fig. 2:**
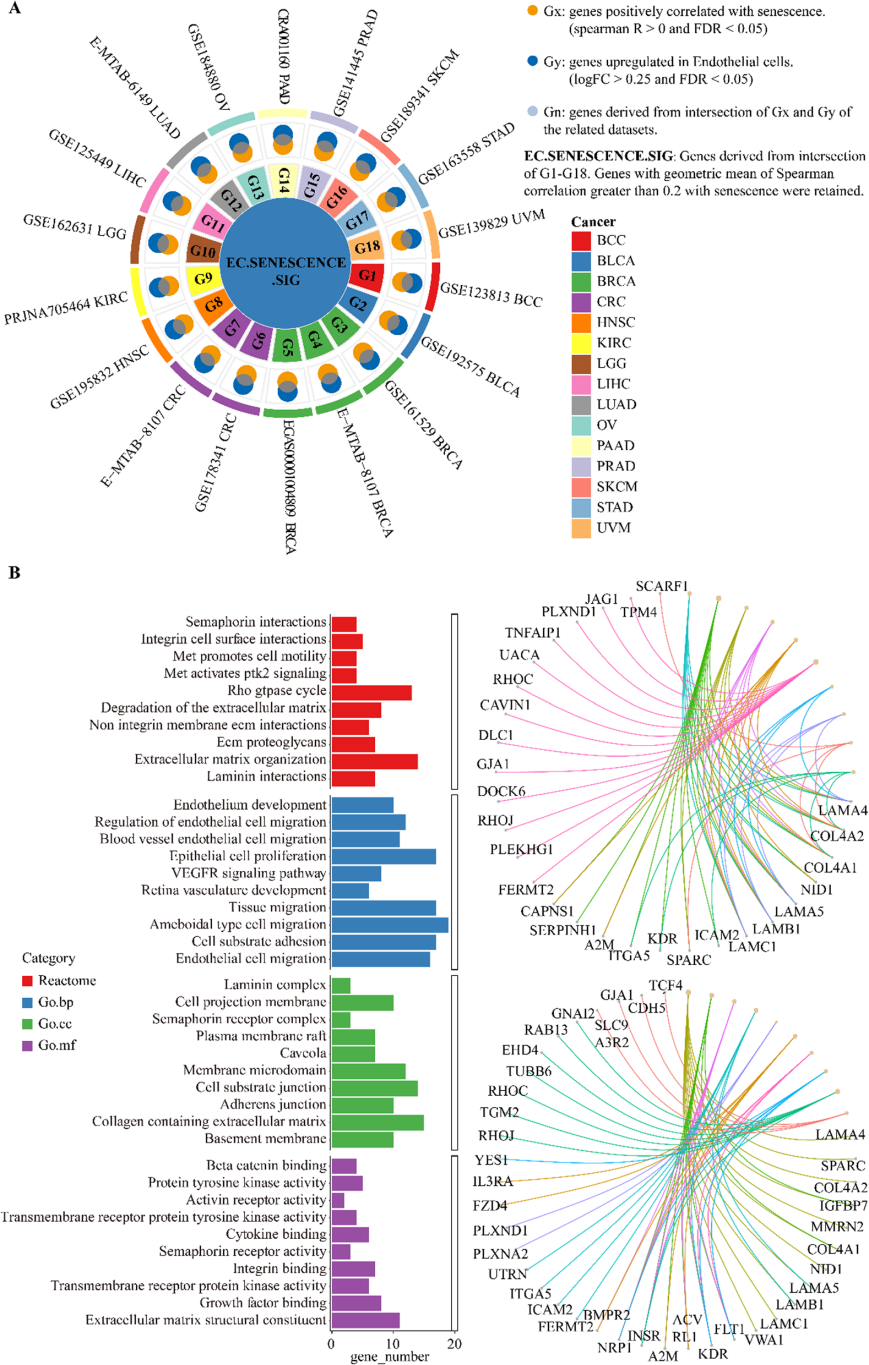
Development of a tumor endothelial cell-specific senescence-related transcriptomic signature through pan‑cancer scRNAseq analysis. **A** The circus diagram shows the generation process of *EC.SENESCENCE.SIG*. **B** (left) Pathway enrichment analysis of *EC.SENESCENCE.SIG* genes. The top 10 enriched GO terms and Reactome pathways are shown in the bar plot. **B** (right) The Cnet plot shows specific gene networks from these signaling pathways

In a next step, we aimed at generating a pan-cancer gene signature that specifically reflects the characteristics of senescent TEC (referred to as *EC.SENESCENCE.SIG*). To this end, eighteen scRNAseq datasets that include fifteen cancer entities were used for Spearman correlation analyses on gene expression levels and GSVA scores (based on *FRIDMAN.SENESCENCE.UP*) of TEC. In these eighteen datasets, genes positively correlated with GSVA scores in TEC (Spearman R > 0 and FDR < 0.05) were considered as ‘Gx’, representing senescence related genes. Genes that were upregulated in endothelial cells (logFC ≥ 0.25 and FDR < 0.05) were considered as ‘Gy’, representing specific endothelial cell genes. To obtain specific endothelial senescence-regulated genes, ‘Gx’ and ‘Gy’ were intersected to generate ‘Gn’ (n = 1–18) for each dataset (Fig. 2A). G1-G18 represent the intersection of the respective Gx and Gy in the 18 scRNA-Seq datasets. Subsequently, the geometric mean of the Spearman correlation coefficients for each gene from ‘G1’ to ‘G18’ were calculated. Finally, only genes with a geometric mean of Spearman correlation coefficient value higher than 0.2 were filtered into *EC.SENESCENCE.SIG*, which ultimately contained 102 genes (Additional file 1: Table S2). To define the functional categories of *EC.SENESCENCE.SIG*, we used two tools for gene annotation in the R ‘clusterProfiler’ package, termed as Gene Ontology (GO) terms and the reactome pathway database [19]. Here, we found that *EC.SENESCENCE.SIG* is mainly enriched by genes associated with cell adhesion- and interaction-related pathways such as ‘integrin cell surface interactions’, ‘laminin interactions’, ‘cell substrate adhesion’, ‘adherens junction’, and ‘Integrin binding’ (Fig. 2B), which is consistent with the previously reported enhanced adhesive properties of senescent endothelial cells [20] and with increased cell–cell communication of HS-TEC with other non-malignant cells.

### Pan-cancer prognostication on signaling pathways, immune cell responses, and patient survival using *EC.SENESCENCE.SIG*

**Fig. 3:**
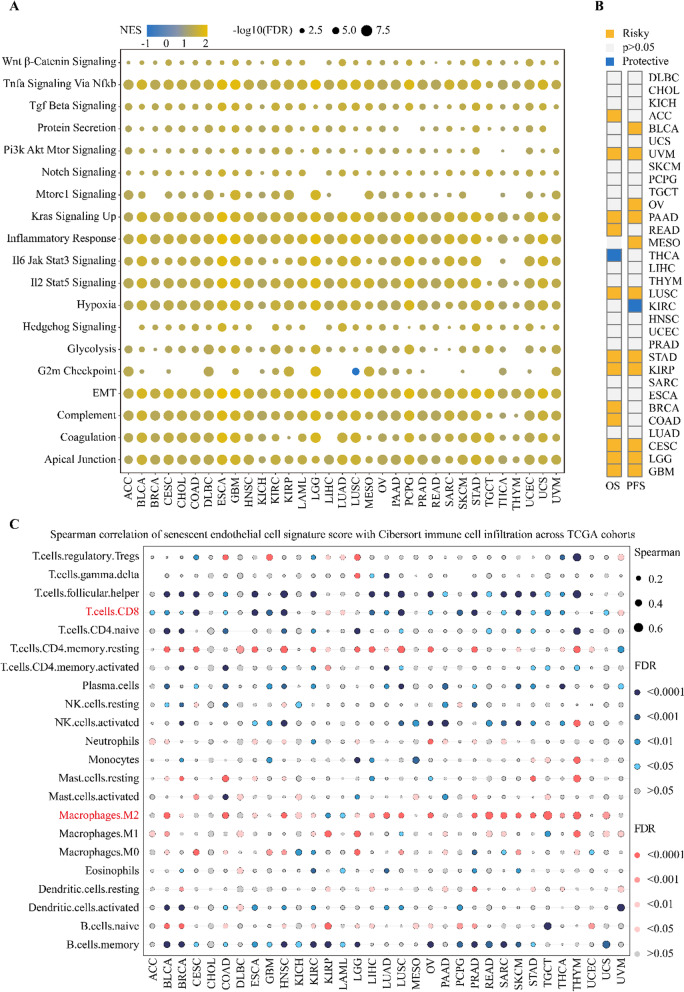
Pan-cancer analysis of *EC.SENESCENCE.SIG*. **A** Enrichment analysis of several tumor-promoting pathways between tumor tissues with high and low *EC.SENESCENCE.SIG* scores across 33 cancer types in TCGA cohorts, *NES* normalized enrichment score in the GSEA algorithm, *FDR* false discovery rates. **B** Summary of the relationship between *EC.SENESCENCE.SIG* scores and OS/PFS of patients across 33 cancer types in TCGA pan-cancer cohorts. Expression of genes associated with poorer prognosis (yellow) or better prognosis (blue) are shown. **C** The correlation of *EC.SENESCENCE.SIG* scores and immune infiltration (Cibersort) across 33 cancer types in TCGA cohorts is shown, blue dots represent a negative correlation, red dots a positive correlation

To further explore the biological characteristics of *EC.SENESCENCE.SIG* across different cancer entities, we applied the GSVA method to *EC.SENESCENCE.SIG* to calculate transcriptomic signature scores for each patient of The Cancer Genome Atlas (TCGA) across thirty-three cancer entities. In general, the results of our analyses indicate that solid malignancies including kidney clear cell carcinoma (KIRC), pancreatic cancer (PAAD), or thyroid cancer (THCA) exhibit higher *EC.SENESCENCE.SIG* scores, whereas hematological malignancies such as large B-cell lymphoma (DLBC) or acute myeloid leukemia (LAML) show lower scores (Additional file 1: Fig. S3). Subdividing patients in TCGA pan-cancer cohorts into high and low GSVA scores (median value) based on *EC.SENESCENCE.SIG*, the enrichment of previously reported tumor-promoting pathways was explored by gene set enrichment analysis (GSEA) for each cancer type. Our results indicate that almost all analyzed pro-tumorigenic signaling pathways were enriched in malignant tumors with high GSVA scores based on *EC.SENESCENCE.SIG* across all cancer types using GSEA (Fig. 3a).

Next, we evaluated the relationship between GSVA score based on *EC.SENESCENCE.SIG* and patient survival in the TCGA cohorts. We found that high GSVA scores were significantly related to impaired overall survival (OS) in more than ten cancer types, including pancreatic cancer (PAAD), lung squamous cell carcinoma (LUSC), stomach cancer (STAD), and kidney papillary cell carcinoma (KIRP). Only in thyroid carcinoma (THCA), patients with high GSVA scores exhibited improved OS as compared to low GSVA scores. In addition, high GSVA scores in eleven cancers correlated with shorter progression free survival (PFS), whereas only in kidney cancer (KIRC) a high GSVA score was associated with enhanced PFS (Fig. 3b). Finally, we used *Cibersort* [17] to evaluate the correlation of GSVA scores (based on *EC.SENESCENCE.SIG*) with the infiltration of twenty-two immune cell subsets across different cancer types in the TCGA cohorts. We found that high GSVA scores are related to distinctly altered tumor infiltration by immune cells. In particular, the GSVA scores in almost all cancer types positively correlated with (pro-tumorigenic) M2 macrophage tumor infiltration and negatively correlated with (anti-tumorigenic) CD8^+^ T cell tumor infiltration (Fig. 3c). In summary, we established an endothelial-specific, senescence-related transcriptomic signature that serves as pan-cancer prognosticator of pro-tumorigenic cell signaling, tumor-promoting dysbalance of immune cell responses, and impaired patient survival.

### Prediction of anti-PD-L1/PD-1 or anti-CTLA-4 immune checkpoint blockade response using *EC.SENESCENCE.SIG*

**Fig. 4:**
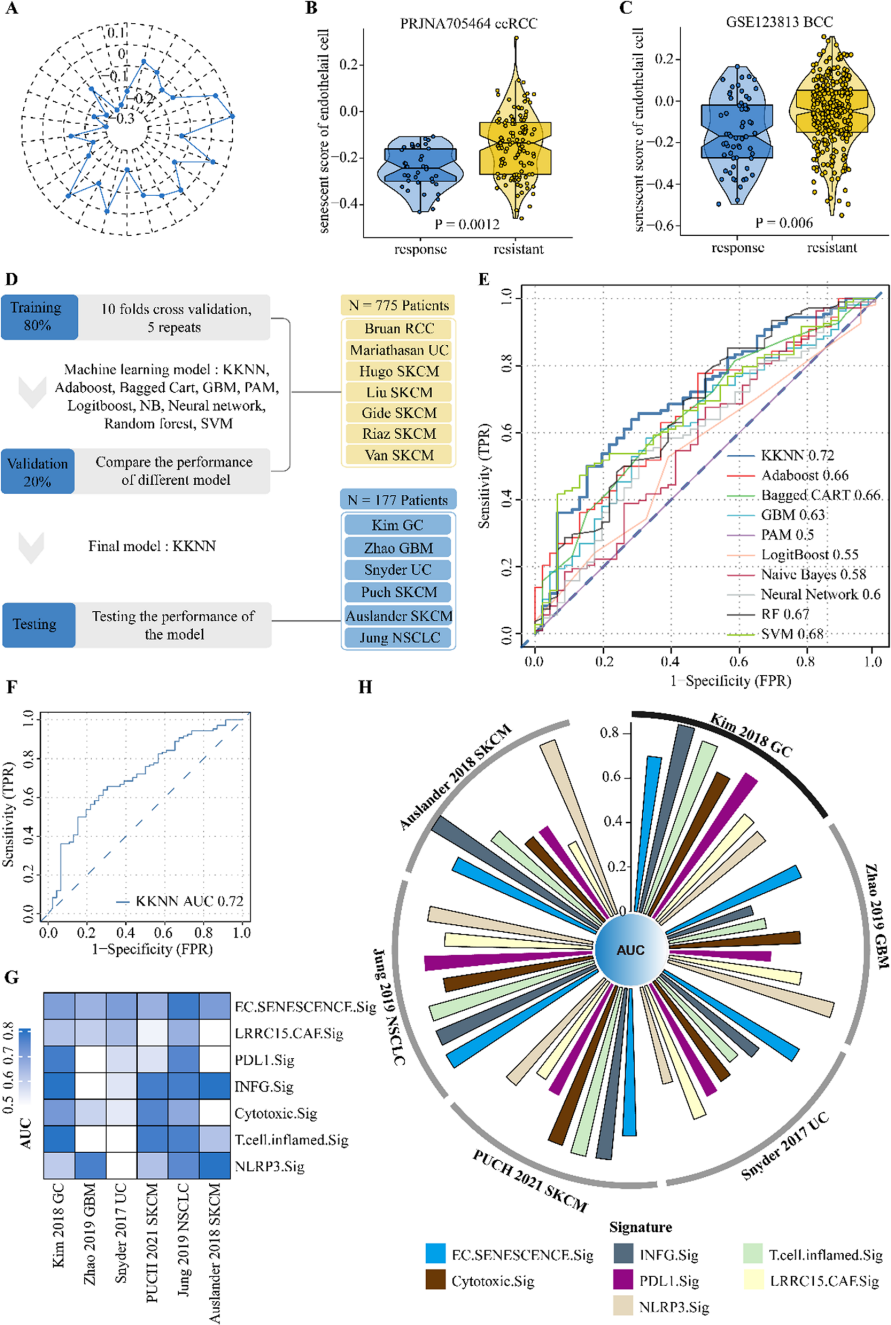
Prediction of outcomes of anti-PD-L1/PD-1 immunotherapy using *EC.SENESCENCE.SIG.* **A** Correlation of *EC.SENESCENCE.SIG* score with TMB for each cancer entity in the pan‑cancer TCGA cohort. **B**, **C** Differences in *EC.SENESCENCE.SIG* scores of TEC derived from patients with different responses to anti-PD-L1/PD-1 immunotherapy, p values are shown. **D** Flow chart describing the construction of machine learning algorithms-based predictive models for immunotherapy response. **E** Multiple receiver operating characteristic (ROC) plot showing the performance of different machine learning algorithms in the validation set. **F** ROC plot presenting the performance of the final *EC.SENESCENCE.SIG* model in the validation set. **G** Heatmap and **H** circus plots show the comparison between the performance of the *EC.SENESCENCE.SIG* model and previously published pan-cancer models for response to anti-PD-L1/PD-1 immunotherapy on different testing sets

With respect to the correlation between high *EC.SENESCENCE.SIG* GSVA scores and a tumor-promoting dysbalance of immune cell infiltration of the tumors, we further hypothesized that this transcriptomic signature also offers the possibility to predict response to anti-PD-L1/PD-1 or anti-CTLA-4 immune checkpoint inhibitor therapy. Tumor mutational burden (TMB) has previously been identified as a robust pan-cancer predictor of anti-PD-L1/PD-1 immunotherapy response [21]. Consequently, we first assessed the correlation between *EC.SENESCENCE.SIG* and TMB in TCGA cohorts. Here, we found that TMB negatively correlates with *EC.SENESCENCE.SIG* in most cancer types (BRCA, HNSC, CESC, LIHC, STAD, MESO, LUSC, KIRP, LUAD, UVM, PRAD, UCEC, and SKCM; Fig. 4a), strongly suggesting that this transcriptomic signature can predict immunotherapy response. Consequently, we investigated the enrichment of *EC.SENESCENCE.SIG* in immunotherapy-responsive and -resistant patients using GSVA and GSEA in three bulk RNAseq datasets with reported clinical outcome of immunotherapy. Here, we found higher GSVA scores in patients resistant to immunotherapy as compared to immunotherapy-responsive patients (Additional file 1: Fig. S4a–c). In line with these results, *EC.SENESCENCE.SIG* was significantly enriched in patients resistant to anti-PD-L1/PD-1 or anti-CTLA-4 immunotherapy in GSEA analyses (Additional file 1: Fig. S4a–c).

In addition to these analyses in bulk RNAseq data, we explored GSVA scores (*EC.SENESCENCE.SIG*) in endothelial cells from two immunotherapy scRNAseq datasets that include renal cancer (PRJNA705464) and basal cell carcinoma (GSE123813) patients. In accordance with our previous findings, endothelial cells derived from immunotherapy-resistant patients exhibited an enrichment of *EC.SENESCENCE.SIG* genes (Fig. 4b, c).

Moreover, we employed thirteen bulk RNAseq datasets containing outcomes of anti-PD-L1/PD-1 or anti-CTLA-4 immunotherapy, of which only treatment-naïve patients were selected for further analyses. Among these thirteen cohorts, seven cohorts (n = 775, 80% for training set, 20% for validation set) were merged as a training cohort, whereas the other six cohorts were used to test the predictive power of the final model developed (Fig. 4d). To this end, we used ten different machine learning algorithms and optimized parameters for each model using five repetitions of tenfold cross-validation. Subsequently, we estimated the area under the curve (AUCs) values of these models in the validation cohort. As a result of these mathematical procedures, we finally choose the ‘KKNN’ machine learning algorithm model that delivered the highest AUC of 0.72 (Fig. 4e, f). Testing the prediction accuracy of this *EC.SENESCENCE.SIG* model in six external cohorts, we show that AUC values in these cohorts range from 0.66 to 0.79 (Additional file 1: Fig. S5).

To estimate the overall value of *EC.SENESCENCE.SIG*-dependent immunotherapy response prediction, we compared the performance of this transcriptomic signature with previously established pan-cancer models for anti-PD-L1/PD-1 or anti-CTLA-4 immunotherapy response prediction, including *NLRP3.Sig* [22], *INFG.Sig* [23], *PDL1.Sig* [24], *T.cell.inflamed.Sig* [25], *Cytotoxic.Sig* [26], and *LRRC15.CAF.Sig* [27]. Whereas most of these pan-cancer prediction models reported good performance only in single datasets, *EC.SENESCENCE.SIG* performed well across all cohorts covering five cancer types including SKCM, GBM, UC, GC, and NSCLC (Fig. 4g, h). In detail, AUC levels of *T.cell.inflamed.Sig* and *INFG.Sig* were around 0.8 in Kim 2018 GC and PUCH 2021 SKCM, but they decreased to around 0.5 in Zhao 2019 GBM and Snyder 2017 UC. *NLRP3.Sig* performed well in Zhao 2019 GBM, Jung 2019 NSCLC, and Auslander 2018 SKCM, whereas it showed poorer performance in the other three cohorts. AUC of *Cytotoxic.Sig* was 0.71 in Kim 2018 GC and 0.75 in PUCH 2021 SKCM, but it went down to 0.54–0.58 in Zhao 2019 GBM and Snyder 2017 UC. AUC of *PDL1.Sig* reached 0.77 in Kim 2018 GC and 0.76 in Jung 2019 NSCLC, but it decreased to 0.45–0.57 in the other four cohorts. *LRRC15.CAF.Sig* showed limited predictive power in all six cohorts. In contrast to these previously published results, *EC.SENESCENCE.SIG* performed well in all cohorts, exhibiting AUC of more than 0.66 in all six cohorts, including five different types of solid cancers (glioblastoma, melanoma, urothelial carcinoma, gastric cancer, and lung cancer). Our results collectively suggest that *EC.SENESCENCE.SIG* also serves as a reliable pan-cancer prediction model for anti-PD-L1/PD1 or anti-CTLA-4 immunotherapy response.

### Construction and validation of a ‘*EC.SENESCENCE.SIG*’-related pan-cancer prognostic model

**Fig. 5:**
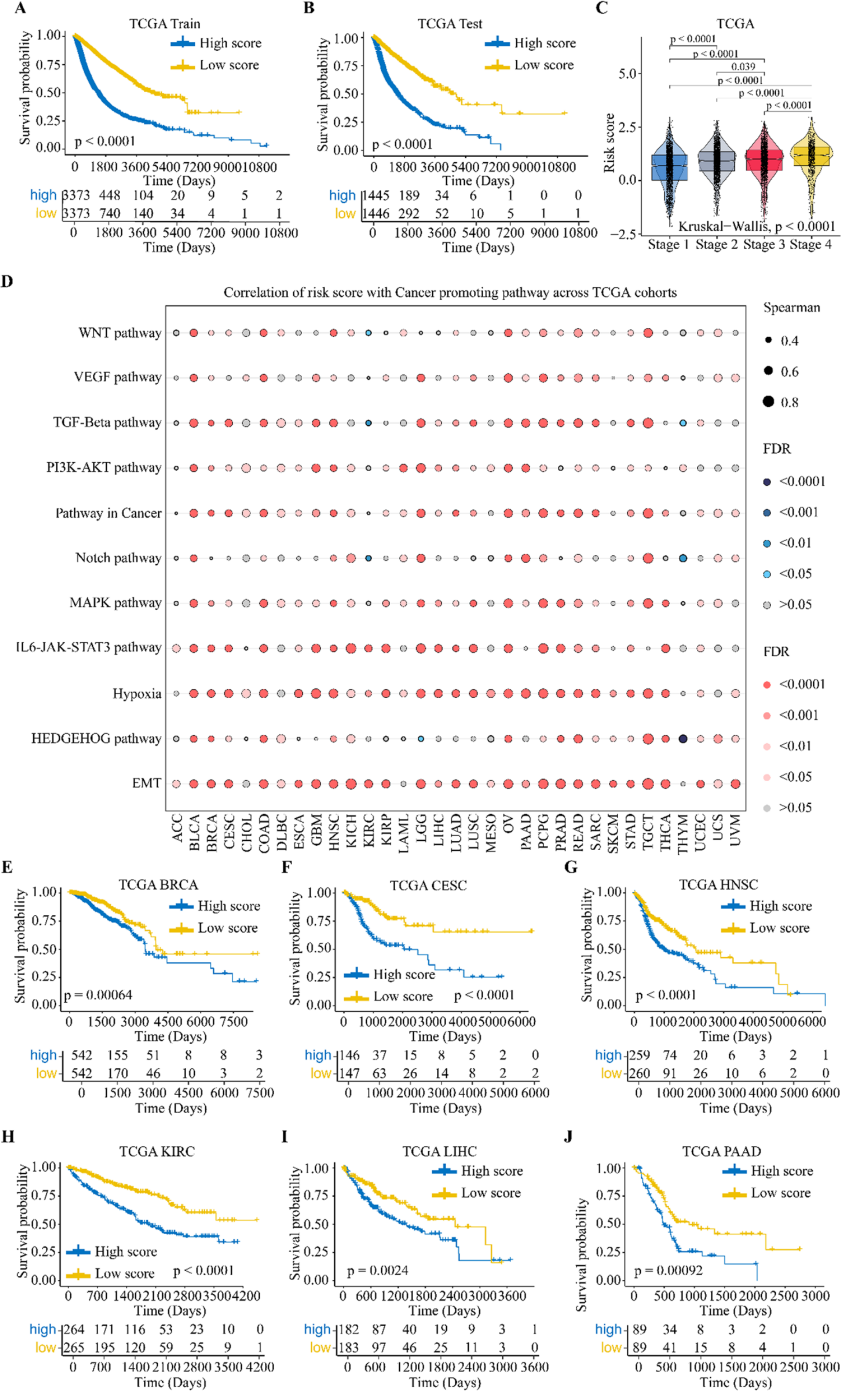
Prognostic performance of *EC.SENESCENCE.SIG*-related pan-cancer model in TCGA. **A**, **B** Kaplan–Meier analysis shows the association between risk score and OS of patients in TCGA pan-cancer training and testing sets. **C** Differences of *EC.SENESCENCE.SIG*-related risk scores between different tumor stages in the TCGA pan-cancer cohort are shown. **D** The correlation of *EC.SENESCENCE.SIG*-related risk score and enrichment of several tumor-promoting pathways across 33 cancer types in TCGA cohorts is shown. Blue dots represent a negative correlation, red dots a positive correlation. **E**–**J** The association between *EC.SENESCENCE.SIG*-related risk score and OS of patients in a variety of tumors is shown, *BRCA* breast invasive carcinoma, *CESC* cervical cancer, *HNSC* head and neck cancer, *KIRC* kidney clear cell carcinoma, *LIHC* liver cancer, *PAAD* pancreatic cancer

To optimize *EC.SENESCENCE.SIG* for pan-cancer survival prognostication, we used this transcriptomic signature to generate a LASSO penalized Cox proportional hazards regression (LASSO-Cox) model. First, the 102 genes of *EC.SENESCENCE.SIG* were included into LASSO analysis with tenfold cross validation in the pan-cancer TCGA cohorts, before fifty genes with non-zero coefficients were identified for further analysis (Additional file 1: Fig. S6a, b). We then employed these fifty genes to develop a Cox proportional hazards regression model using a stepwise parameter selection method in the TCGA pan-cancer training set (Additional file 1: Fig. S6c). The risk score for each patient was subsequently developed from the Cox coefficients and normalized expression levels of these thirty-seven genes. Finally, we classified the patients in the TCGA training test sets into two groups according to the median value of the risk score. Here, we found that patients with higher risk scores in both cohorts were associated with worse overall survival (Fig. 5a, b). Accordingly, patients with higher clinical stage had a significantly higher risk score (Fig. 5c). We subsequently calculated the correlation coefficients between *EC.SENESCENCE.SIG*-related risk score and GSVA score of several selected tumor-promoting signaling pathways. Interestingly, our risk score positively correlated with all these pathways’ GSVA scores across all cancer types in the TCGA cohorts (Fig. 5d).

**Fig. 6:**
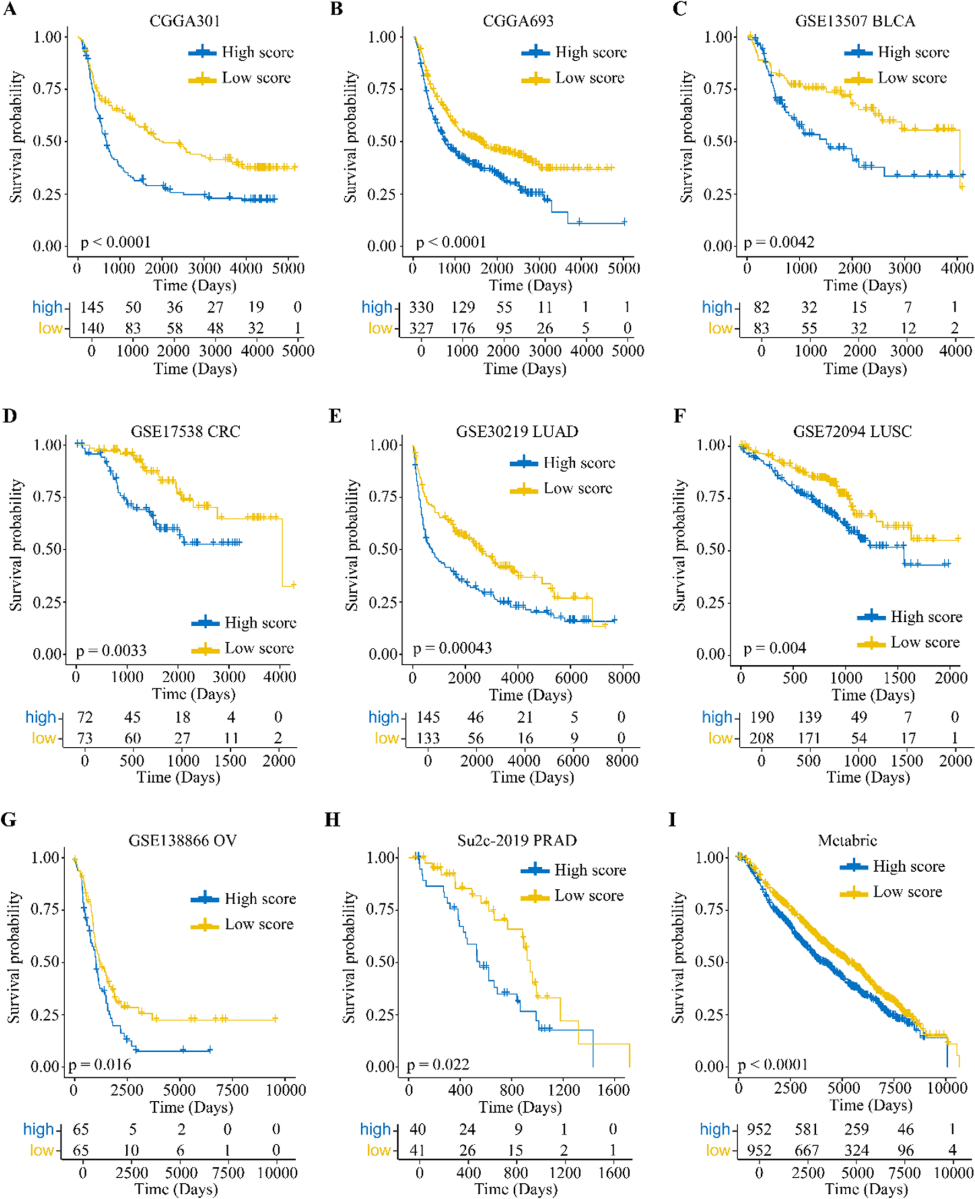
Prognostic performance of the *EC.SENESCENCE.SIG*-related pan-cancer model in external cohorts.** A**–**I** Kaplan–Meier analysis on the association between risk score and OS of patients in external cohorts, p values are given. *CGGA* Chinese Glioma Genome Atlas, *METABRIC* Molecular Taxonomy of Breast Cancer International Consortium, *BLCA* bladder cancer, *CRC* colon cancer, *LUAD* lung adenocarcinoma, *LUSC* lung squamous cell carcinoma, *OV* ovarian cancer, *PRAD* prostate cancer

We also observed that the *EC.SENESCENCE.SIG*-related risk score demonstrates strong prognostic power for overall survival of various other cancer types, including BRCA (log rank test: P = 0.00064), cervical cancer (CESC, log rank test: P < 0.0001), HNSC (log-rank test: P < 0.0001), KIRC (log rank test: P < 0.0001), LIHC (log rank test: P = 0.0024), and PAAD (log rank test: P = 0.00092; Fig. 5e–j). To further confirm the prognostic value of this risk score, we calculated the risk score using the same formula in several external validation cohorts. These *EC.SENESCENCE.SIG*-related risk scores also show good performance in prognosticating patient survival in these datasets (Fig. 6a–i), indicating that our risk score is a reliable prognosticator in a variety of cancers.

### Establishment of ‘*EC.SENESCENCE.SIG*’-related risk score-based nomogram for clinical prognostication of pan-cancer survival

**Fig. 7:**
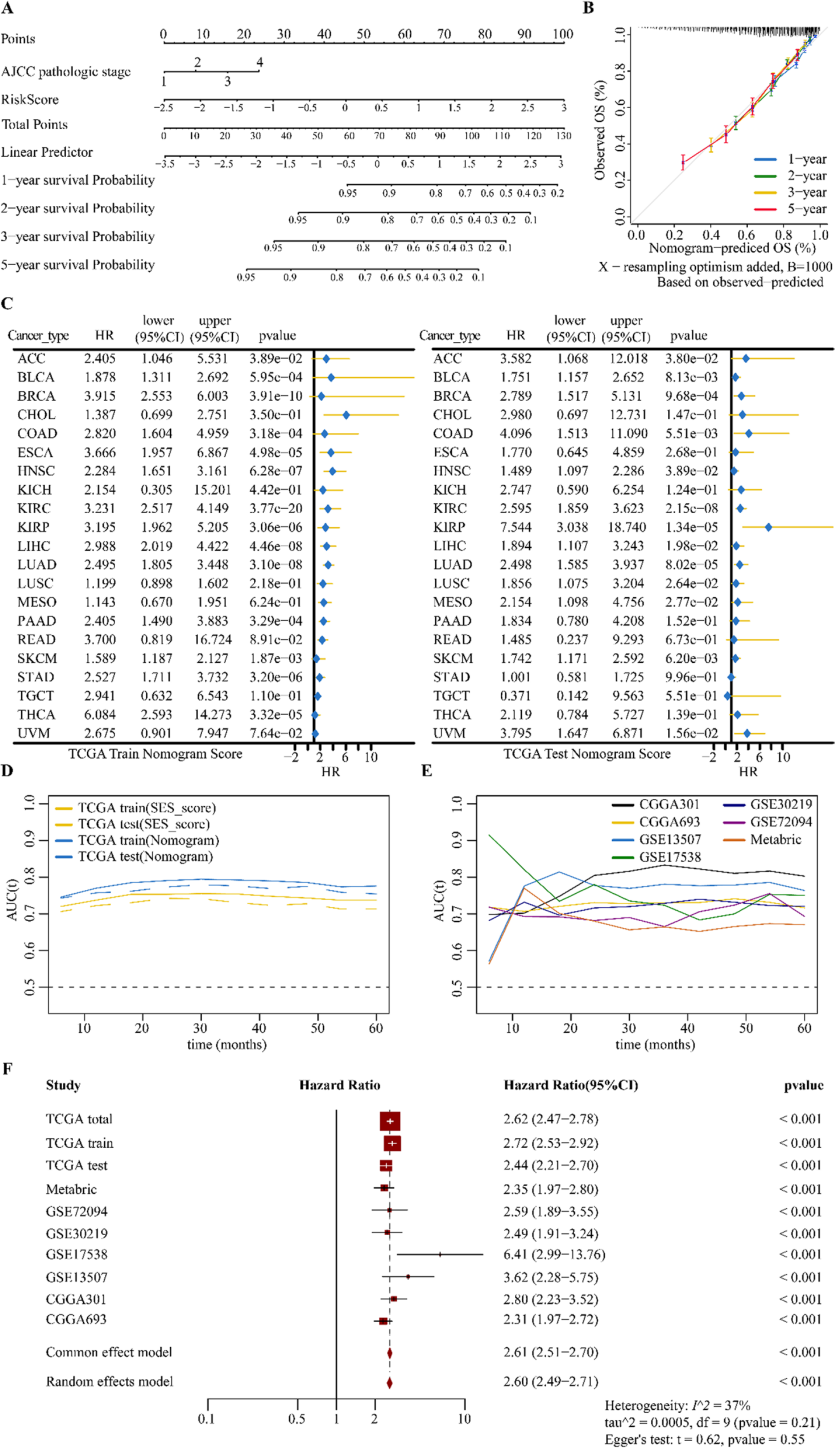
Effectiveness assessment of *EC.SENESCENCE.SIG*-derived nomogram features in predicting pan-cancer prognosis. **A** Nomograms for predicting overall survival of patients in TCGA pan-cancer cohort are shown. **B** The calibration of our model to ensure consistency between predictions and real survival are shown, the 45-degree line represents perfect prediction. **C** The univariate Cox analysis of the nomogram score for different cancer types in pan-cancer TCGA training and testing sets. **D** Time-dependent ROC curves show the prognostic performance of the *EC.SENESCENCE.SIG*-related score and nomogram score in pan-cancer TCGA training and testing sets. **E** Time-dependent ROC curves show the prognostic performance of the *EC.SENESCENCE.SIG*-related score and nomogram score in external cohorts. **F** Meta-analysis of the prognostic performance of nomogram score in these ten cohorts

To further strengthen the prognostic power of the risk score developed above, we generated a nomogram score combining the clinical disease stage with the *EC.SENESCENCE.SIG*-related risk score in the TCGA pan-cancer cohorts (Fig. 7a). The calibration curves of disease-specific survival (DSS) in the first five years after cancer diagnosis showed that the prognosticated survival probability is highly consistent with actual survival, indicating the robustness of this nomogram in survival prognostication (Fig. 7b). Furthermore, univariate Cox analysis describing the effect of the nomogram score on overall survival of TCGA pan-cancer cohorts demonstrated that the nomogram score is associated with impaired survival in most types of cancer (Fig. 7c). Importantly, the time-dependent AUC predicted by the nomogram score performed better than the *EC.SENESCENCE.SIG*-related risk score alone in both the TCGA training set and the test cohort (Fig. 7d and Additional file 1: Fig. S7a, b). Moreover, this nomogram score demonstrated promising prognostic performance in external validation datasets with different cancer entities (Fig. 7e). Finally, we conducted a prognostic meta-analysis to examine the combined prognostic value of these ten training and validation sets. Here, the nomogram score serves as a significant risk factor for overall survival in cancer patients (combined HR = 2.61, P < 0.001; Fig. 7f).

### Prognostic feature selection of *EC.SENESCENCE.SIG*

To facilitate the clinical application of *EC.SENESCENCE.SIG* in the evaluation of survival prognosis, we used three machine learning-based algorithms including Random Forest, extreme gradient boost (XGBoost), and LASSO feature selection to select the most important signatures from all genes in *EC.SENESCENCE.SIG*. For the prognostication of overall survival in the TCGA pan-cancer cohorts, we identified fifty genes by LASSO, nine by random forest, and nine by XGBoost (Additional file 1: Fig. S8a, b). Subsequently, we took an intersection and obtained three common genes, including *integrin subunit alpha 5* (ITGA5), *transglutaminase 2* (TGM2), and *fascin actin-bundling protein 1* (FSCN1) (Additional file 1: Fig. S8c). Next, we analyzed the differential expression of these three genes between tumors and normal tissues across twenty cancer types of the TCGA cohorts. Of these genes, FSCN1 was upregulated across all cancer types as compared to normal tissues, whereas ITGA5 and TGM2 were only upregulated in 70% of the tumors (Additional file 1: Fig. S8d). Finally, we focused on the association between these three genes and survival prognosis of patients across the thirty-three cancer types. Here, we found that high expression of FSCN1 and ITGA5 is associated with impaired survival in more than ten cancers, and high expression of TGM2 is also associated with poor prognosis in more than five cancers (Additional file 1: Fig. S8e). Altogether, our results suggest that these three hub genes from *EC.SENESCENCE.SIG* might serve as prognostic pan-cancer biomarkers.

## Discussion

Individual prognostication of patient survival and prediction of response to therapy are critical for the development of personalized treatment strategies in precision oncology. Microvascular endothelial cells substantially participate in nutrient and oxygen delivery to malignant tumors as well as in immune surveillance [28]. Specifically, tumor-associated endothelial cells can produce molecular factors such as PD-L1 [29], Fas ligand (FasL) [30, 31], or vascular endothelial growth factor (VEGF) [32] that critically modulate immune responses in the tumor environment. Cellular senescence considerably modulates the functional properties of cells and has recently been attributed to the ‘hallmarks of cancer’ as a key feature of solid malignancies [3]. To this end, senescent cancer cells recruit immunosuppressive immune cells by the release of diverse molecular factors [33–35]. Interestingly enough, senescence in cancer cells has also been reported to activate CD8^+^ T cells through release of alarmins, activation of interferon signaling, upregulation of major histocompatibility complex (MHC) class I machinery, and presentation of senescence-associated self-peptides, ultimately promoting the elimination of tumor cells [36, 37]. These inconsistent findings highlight the multifaceted role of cellular senescence in cancer biology. Besides tumor cells, cells in the tumor microenvironment including fibroblasts, immune cells, and endothelial cells undergo senescence-related alterations, which critically contributes to tumor progression: senescent cancer-associated fibroblasts support tumor growth and invasion through the secretion of cytokines and extracellular vesicles [15, 38–40]. Furthermore, it has been demonstrated that senescence in T cells substantially impairs their potential to eliminate cancer cells [41, 42]. In addition, previous studies have shown that senescent endothelial cells exhibit enhanced expression levels of *intercellular adhesion molecule-1* (ICAM-1) and *vascular cell adhesion molecule-1* (VCAM-1) as well as reduced expression of *vascular endothelial* (VE)-*cadherin*, which leads to increased vascular permeability and facilitates the dissemination of cancer cells [43–45]. Moreover, chemotactic cytokines such as IL-6 or CXCL11 secreted by senescent endothelial cells can directly stimulate proliferation and aggressiveness of tumor cells [46]. Importantly, all these molecular factors in endothelial cells additionally bear the potential to recruit immune cells into the tumor microenvironment, which can exhibit both anti- and pro-tumorigenic properties [45, 47–49]. Finally, induction of endothelial cell senescence by radiotherapy stimulated tumor cell proliferation and cancer progression [45, 49]. Consequently, senescence of TEC might serve as a promising target for survival prognostication and prediction of immunotherapy response in cancer, and possibly as therapeutic target.

To address the prognostic and predictive capacity of senescence in TEC, we first conducted analyses in public scRNAseq cohorts evaluating the degree of cellular senescence in individual cell populations of solid tumors across multiple cancer entities. For this purpose, we employed an established senescence-related gene signature [16] that—in contrast to other senescence-related gene signatures [50, 51]—more universally covers senescence-related transcriptomic changes. Our transcriptomic studies reveal that TEC exhibit the highest senescence levels among all cell populations in the vascular compartment of malignant tumors. Since tumor microvessels are highly disorganized and show abnormal functional properties including aberrant immune cell interactions [31, 32], cellular senescence might particularly contribute to these pathological TEC characteristics. Using network analysis and pattern recognition approaches, we show that HS-TEC frequently establish interactions with different cell types, which are typically represented by immune cells, as opposed to TEC with low levels of cellular senesce. Interestingly, this interplay particularly involves immune cells with pro-tumorigenic properties such as myeloid cells. For this purpose, HS-TEC were identified to secrete the cytokine MIF that is capable of inducing myeloid-derived suppressor cell (MDSC) proliferation [52], promoting M2 macrophage polarization [53], and impairing CD8^+^ T cell infiltration [54]. Furthermore, HS-TEC were found to release galectins, which represent carbohydrate-binding proteins known to support immune cell migration [55–57]. Hence, our results suggest that TEC are prone to undergo cellular senescence, which promotes dysregulated immune surveillance in solid malignancies.

Based on these findings, we developed a novel, TEC-specific transcriptomic signature containing 102 genes, which we have termed *EC.SENESCENCE.SIG*. Mechanistically, these genes are mainly related to the regulation of cell adhesion (e.g., intercellular adhesion molecule-2, laminins). This is in accordance with previous reports, documenting that senescent endothelial cells exhibit enhanced adhesive properties mediated by increased expression of such adhesion and signaling molecules [20].

In a next step, we evaluated the relationship of our transcriptomic signature to the prevalence of specific signaling pathways, the occurrence of distinct immune cell responses, and patient survival. We found that *EC.SENESCENCE.SIG* positively correlates with a variety of pro-tumorigenic signaling pathways in all cancer entities of the TCGA cohorts. Among these pro-tumorigenic signaling pathways, hypoxia and Notch signaling pathways have not only been shown to be associated with tumor progression, but also represent important regulators of endothelial cell senescence [45, 58]. In addition, *EC.SENESCENCE.SIG* correlates with dysregulated tumor infiltration by CD8^+^ T cells and M2-polarized tumor-associated macrophages, which is considered a determinant of anti-PD-L1/PD-1 immunotherapy efficacy [59, 60]. In line with these results, high *EC.SENESCENCE.SIG* GSVA scores were associated with impaired OS and PFS in more than ten different cancer entities.

With respect to the immunomodulatory potential of senescent TEC, our TEC-based, senescence-related gene signature might also predict response to cancer immunotherapy. Here, we demonstrate in the TCGA cohorts that *EC.SENESCENCE.SIG* negatively correlates with TMB, which to date represents one of the most robust predictive biomarkers of response to anti-PD-L1/PD-1 immune checkpoint blockade [21]. Accordingly, *EC.SENESCENCE.SIG* predicts response to anti-PD-L1/PD-1 or anti-CTLA-4 immunotherapy in multiple cancer entities including lung cancer, gastric cancer, urothelial carcinoma, renal cell carcinoma, and basal-cell carcinoma. In this context, *EC.SENESCENCE.SIG* shows promising AUC values in different datasets, thus providing higher performance than previously published pan-cancer predictive models for immunotherapy efficacy which only reported sufficient performance in few datasets [22–27].

To strengthen the prognostic potential of our model, we additionally established a nomogram based on LASSO-Cox regression. Here, we found that high risk scores positively correlate with higher disease stage and with impaired OS in pan-cancer cohorts. This was associated with an enrichment of pro-tumorigenic signaling pathways in almost all cancer entities of the TCGA cohorts. To further improve the accuracy of this prognostic model, we combined the clinical disease stage with this *EC.SENESCENCE.SIG*-related risk score. Here, we show that survival prognostication based on this nomogram score is highly consistent with the actual survival probability during a follow-up period of 5 years.

To facilitate the clinical application of *EC.SENESCENCE.SIG*, we identified the integrin ITGA5, transglutaminase TGM2, and the actin-binding protein FSCN1 as key genes of *EC.SENESCENCE.SIG* for prognosticating OS. Interestingly, EC senescence along with ITGA5 surface translocation can be induced by hemodynamic forces [20, 61], which are known to be profoundly altered in growing tumor vessels. Here, ITGA5 is able to promote tumor metastasis and drug resistance via activating extracellular signal-regulated kinases (Erk)1/2 [62, 63]. In addition to EC, ITGA5 is over-expressed in numerous carcinoma entities and is an integral part of the pEMT signature of single malignant cells in HNSCC [64]. Therefore, ITGA5 might provide dual options to target senescent TEC as well as malignant carcinoma cells associated with the formation of lymph node metastases. Furthermore, TGM2 is involved in age-related kidney and cardiovascular diseases and upregulated in vascular endothelial cells in gastrointestinal cancer, which is associated with poor patient survival via activating downstream NF-κB-dependent pathways [65, 66]. Finally, FSCN1 has been reported to be overexpressed in various cancer entities including lung and breast cancer and to promote metastasis formation in colon, prostate, and oral squamous cell carcinoma by mediating the formation of filopodia and membrane protrusions [67, 68].

Importantly, there are several limitations in our study. First, the results of our retrospective pan-cancer data analyses must be validated by multiple prospective trials to exclude potential selection or misclassification bias. In addition, this study exclusively relies on transcriptomic data from published cohorts that do not necessarily translate into protein functionality. Analyses of regulatory network formation using NetBid2 or of interference of protein activity using metaVIPER, however, are only possible in selected carcinoma entities. Moreover, large-scale pan-cancer cohorts with proteomic data are currently missing. Our studies should therefore be accordingly extended when access to such proteomic databases is available.

## Conclusions

In summary, our studies provide novel insights into various molecular and cellular processes associated with cellular senescence in the vascular compartment of malignant tumors. As a translational perspective, the endothelial cell-associated, senescence-related pan-cancer gene signature *EC.SENESCENCE.SIG* established in the present study might be beneficial for survival prognostication and prediction of response to immunotherapy in precision oncology.

## Supplementary Information

**Additional file 1: Table S1.** Gene list of *FRIDMAN_SENESCENCE_UP*. A list of genes forming the *FRIDMAN_SENESCENCE_UP* signature is shown. **Table S2.** Gene list of *EC.SENESCENCE.SIG*. A list of genes forming the *EC.SENESCENCE.SIG* signature is shown. **Table S3.** Immunotherapy cohorts. A list of immunotherapy cohorts used in this study is shown. **Figure S1.** Cell interaction analysis using CellChat. Circle plots show the cellular interaction weights and number of interactions between high-senescent (HS-TEC), low-senescent tumor endothelial cells (LS-TEC) and other cell types in tumor microenvironment in lung cancer (A), liver cancer (B) and head and neck cancer (C). Different colors in the circle plots represent different cell types and the edge width is proportional to the indicated cell–cell interaction weights. **Figure S2.** Analysis of signaling pathways involved in cell–cell interactions. Heatmaps show the outgoing (left) and incoming (right) signal strength of each signaling pathway among different cell types in lung cancer (A), liver cancer (B) and head and neck cancer (C). Bubble plots show all significant ligand-receptor pairs that contribute to the signaling sending from high-senescent tumor endothelial cells (HS-TEC) to other cell types in lung cancer (A), liver cancer (B) and head and neck cancer (C). The dot color and size in the bubble plot represent the communication probability and p-values, with blue and red corresponding to the minimum and maximum values, respectively. **Figure S3.** *EC.SENESCENCE.SIG* gsva score across 33 cancer types in TCGA pan-cancer cohorts. **Figure S4.** Performance of the *EC.SENESCENCE.SIG*-dependent pan-cancer predictive model. (A-C) The upper plots show the difference of *EC.SENESCENCE.SIG* scores in response and resistance to checkpoint immunotherapy groups. The differences were calculated by Wilcoxon rank sum test. The lower plots show the positive enrichment of *EC.SENESCENCE.SIG* in patients with resistance to checkpoint immunotherapy in lung cancer (GSE135222), gastric cancer (Kim GC) and urothelial carcinoma (Mariathasan UC) respectively. NES: Normalized enrichment score in the GSEA algorithm. **Figure S5.** Performance of the *EC.SENESCENCE.SIG*-dependent pan-cancer prognostic model. Receiver operating characteristic (ROC) plots show the performance of the *EC.SENESCENCE.SIG* in distinguishing response and resistant to immunotherapy in six different cohorts. Area Under Curve (AUC) was calculated by ROC analysis and are displayed in the bottom right. **Figure S6.** Construction of a *EC.SENESCENCE.SIG*-related pan-cancer prognostic model (**A**, **B**) LASSO coefficient profiles of the 50 selected genes in EC.SENESCENCE.SIG. tenfold cross-validation to select tuning parameters for the LASSO model. (**C**) Forest plot of a multivariate Cox proportional hazards regression model in the overall survival of TCGA pan-cancer cohort. **Figure S7.** Performance of the *EC.SENESCENCE.SIG*-dependent pan-cancer prognostic model. (A) Receiver operating characteristic (ROC) plots show the performance of the *EC.SENESCENCE.SIG*-related pan-cancer prognostic model in predicting overall survival of pan-cancer TCGA training and test cohorts. (B) ROC plots show the performance of the nomogram model in predicting overall survival of pan-cancer TCGA training and test cohorts. Area Under Curve (AUC) at 12 months, 24 months, 36 months and 60 months were calculated by ROC analysis and are displayed in the bottom right. **Figure S8.** Prognostic feature selection of *EC.SENESCENCE.SIG*. Importance values of selected genes in *EC.SENESCENCE.SIG* for patient prognosis assessment using random forest (**A**) or XGboost (**B**). (**C**) The flow chart shows the selection process of the three key genes in *EC.SENESCENCE.SIG* to predict OS of patients in the TCGA pan-cancer cohort. (**D**) Differential expression of these 3 genes in tumor tissue relative to normal tissue among 20 cancer types in the pan-cancer TCGA cohort. X-fold changes as compared to normal tissue are shown. (**E**) A summary of the relationship between expression of these 3 hub genes and patient prognosis (OS and PFS) across 33 cancer types in the TCGA pan-cancer cohort is shown.

## Data Availability

All the datasets could be downloaded directly from the indicated websites. Datasets and custom scripts are available upon request.
