# Supplementary material for: A transcriptomic pan-cancer signature for survival prognostication and prediction of immunotherapy response based on endothelial senescence
Source: J Biomed Sci. 2023 Mar 28;30:21. doi: 10.1186/s12929-023-00915-5 (PMC10045484; doi:10.1186/s12929-023-00915-5)
Supplement: Supplementary file 1 — Additional file 1: Table S1. Gene list of FRIDMAN_SENESCENCE_UP. A list of genes forming the FRIDMAN_SENESCENCE_UP signature is shown. Table S2. Gene list of EC.SENESCENCE.SIG. A list of genes forming the EC.SENESCENCE.SIG signature is shown. Table S3. Immunotherapy cohorts. A list of immunotherapy cohorts used in this study is shown. Figure S1. Cell interaction analysis using CellChat. Circle plots show the cellular interaction weights and number of interactions between high-senescent (HS-TEC), low-senescent tumor endothelial cells (LS-TEC) and other cell types in tumor microenvironment in lung cancer (A), liver cancer (B) and head and neck cancer (C). Different colors in the circle plots represent different cell types and the edge width is proportional to the indicated cell–cell interaction weights. Figure S2. Analysis of signaling pathways involved in cell–cell interactions. Heatmaps show the outgoing (left) and incoming (right) signal strength of each signaling pathway among different cell types in lung cancer (A), liver cancer (B) and head and neck cancer (C). Bubble plots show all significant ligand-receptor pairs that contribute to the signaling sending from high-senescent tumor endothelial cells (HS-TEC) to other cell types in lung cancer (A), liver cancer (B) and head and neck cancer (C). The dot color and size in the bubble plot represent the communication probability and p-values, with blue and red corresponding to the minimum and maximum values, respectively. Figure S3. EC.SENESCENCE.SIG gsva score across 33 cancer types in TCGA pan-cancer cohorts. Figure S4. Performance of the EC.SENESCENCE.SIG-dependent pan-cancer predictive model. (A-C) The upper plots show the difference of EC.SENESCENCE.SIG scores in response and resistance to checkpoint immunotherapy groups. The differences were calculated by Wilcoxon rank sum test. The lower plots show the positive enrichment of EC.SENESCENCE.SIG in patients with resistance to checkpoint immunotherapy in lung canc [file 12929_2023_915_MOESM1_ESM.docx]

**Additional Tables**

**Table S1. Gene list of *FRIDMAN_SENESCENCE_UP*.** A list of genes forming the *FRIDMAN_SENESCENCE_UP* signature is shown.

| **HSPA2** | **CDKN2A** | **SERPINE1** | **CDKN2B** | **CYP1B1** | **CCND1** | **RRAS** | **RHOB** | **FILIP1L** | **NRG1** |
| --- | --- | --- | --- | --- | --- | --- | --- | --- | --- |
| **RAB31** | **CCN2** | **VIM** | **IGFBP4** | **MMP1** | **RAB5B** | **S100A11** | **GUK1** | **MAP2K3** | **MAP1LC3B** |
| **CXCL14** | **IRF5** | **CITED2** | **HTATIP2** | **CDKN2D** | **IGFBP3** | **NME2** | **ISG15** | **NDN** | **IGFBP2** |
| **RBL2** | **TSPYL5** | **CLTB** | **IRF7** | **IGFBP7** | **F3** | **IGFBP6** | **TNFAIP3** | **TP53** | **IGFBP5** |
| **HBS1L** | **ALDH1A3** | **RAC1** | **STAT1** | **IFNG** | **IGSF3** | **THBS1** | **IFI16** | **ING1** | **CDKN1C** |
| **OPTN** | **RGL2** | **CREG1** | **SOD1** | **CRYAB** | **COL1A2** | **HPS5** | **RABGGTA** | **SMURF2** | **PEA15** |
| **AOPEP** | **RAB13** | **MDM2** | **TFAP2A** | **TGFB1I1** | **SPARC** | **TNFAIP2** | **SERPINB2** | **TES** | **CD44** |
| **IGFBP1** | **FN1** | **CDKN1A** | **EIF2S2** | **ESM1** | **SMPD1** | **GSN** |  |  |  |

**Table S2. Gene list of *EC.SENESCENCE.SIG*.** A list of genes forming the *EC.SENESCENCE.SIG* signature is shown.

| **SLC9A3R2** | **FERMT2** | **PLXNA2** | **FLT1** | **CAV2** | **ICAM2** | **GALNT18** | **LAMA4** | **SPARC** | **PCDH12** |
| --- | --- | --- | --- | --- | --- | --- | --- | --- | --- |
| **PLEKHG1** | **MYCT1** | **EFNB2** | **CD93** | **RHOJ** | **KDR** | **PODXL** | **DLL4** | **DOCK6** | **PLVAP** |
| **TMEM204** | **NES** | **COL4A2** | **HECW2** | **DUSP6** | **ACVRL1** | **PTPRG** | **ESAM** | **PRSS23** | **GJA1** |
| **AFAP1L1** | **STC1** | **COX7A1** | **ITGA5** | **BCL6B** | **IGFBP7** | **TM4SF18** | **DLC1** | **JCAD** | **CYYR1** |
| **SYNPO** | **MMRN2** | **CD34** | **FZD4** | **A2M** | **CAVIN1** | **CDH5** | **IL3RA** | **BCAM** | **COL4A1** |
| **S100A16** | **TCF4** | **TGM2** | **BMPR2** | **SCARF1** | **ECE1** | **PLK2** | **RHOC** | **SERPINH1** | **INSR** |
| **IPO11** | **MAGI1** | **NID1** | **MECOM** | **UACA** | **TUBB6** | **LMO2** | **NECTIN2** | **GRB10** | **LAMA5** |
| **LUZP1** | **MAST4** | **DYSF** | **PNP** | **NRP1** | **CAVIN3** | **LRRC8A** | **EFNA1** | **NFIA** | **EHD4** |
| **TNFAIP1** | **PLXND1** | **LAMB1** | **RGS3** | **ZEB1** | **TRIOBP** | **FSCN1** | **YES1** | **JAG1** | **PEA15** |
| **RAB13** | **PHACTR2** | **LAMC1** | **VWA1** | **PPIC** | **SLC44A2** | **PLEKHA1** | **TPM4** | **GNAI2** | **MGLL** |
| **UTRN** | **CAPNS1** |  |  |  |  |  |  |  |  |

**Table S3. Immunotherapy cohorts.** A list of immunotherapy cohorts used in this study is shown.

| Cohorts | Cancer | Number (patients) | PMID |
| --- | --- | --- | --- |
| Hugo SKCM^1^ | Melanoma | 26 | 26997480 |
| Liu SKCM^2^ | Melanoma | 121 | 31792460 |
| Gide SKCM^3^ | Melanoma | 73 | 30753825 |
| Riaz SKCM^4^ | Melanoma | 49 | 29033130 |
| Van SKCM^5^ | Melanoma | 36 | 26359337 |
| PUCH SKCM^6^ | Melanoma | 49 | 33542239 |
| Auslander SKCM^7^ | Melanoma | 14 | 30127394 |
| Mariathasan UC^8^ | Urothelial carcinoma | 298 | 29443960 |
| Snyder UC^9^ | Urothelial carcinoma | 25 | 28552987 |
| Zhao GBM^10^ | Glioma | 17 | 30742119 |
| Kim GC^11^ | Gastric cancer | 45 | 30013197 |
| Jung NSCLC^12^ | Lung cancer | 27 | 31537801 |
| Bruan RCC^13^ | Renal cell carcinoma | 172 | 32472114 |
| Total |  | 952 |  |

**Additional figures**

**
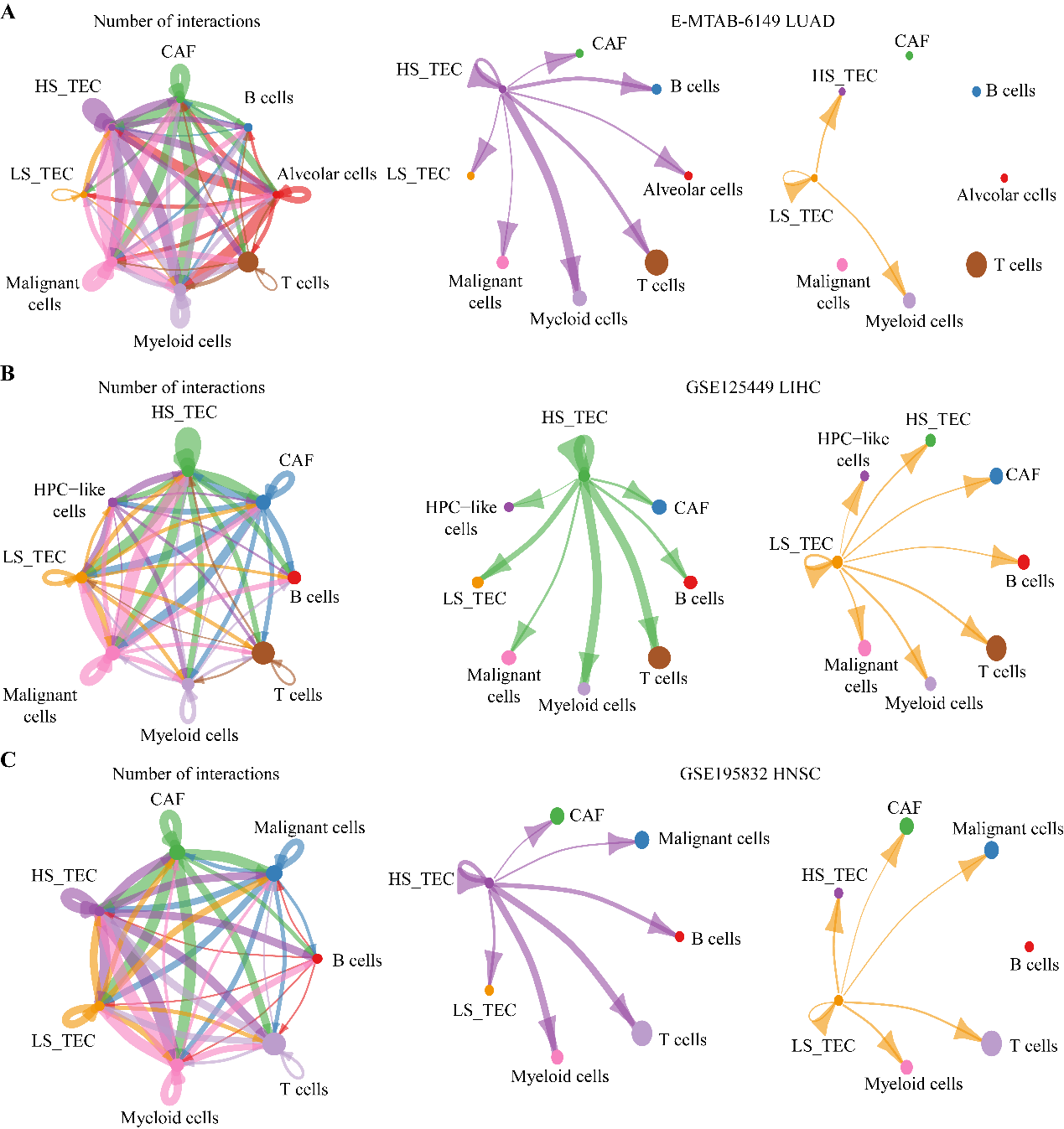
**

**Figure S1. Cell interaction analysis using CellChat.** Circle plots show the cellular interaction weights and number of interactions between high-senescent (HS-TEC), low-senescent tumor endothelial cells (LS-TEC) and other cell types in tumor microenvironment in lung cancer (A), liver cancer (B) and head and neck cancer (C). Different colors in the circle plots represent different cell types and the edge width is proportional to the indicated cell-cell interaction weights.


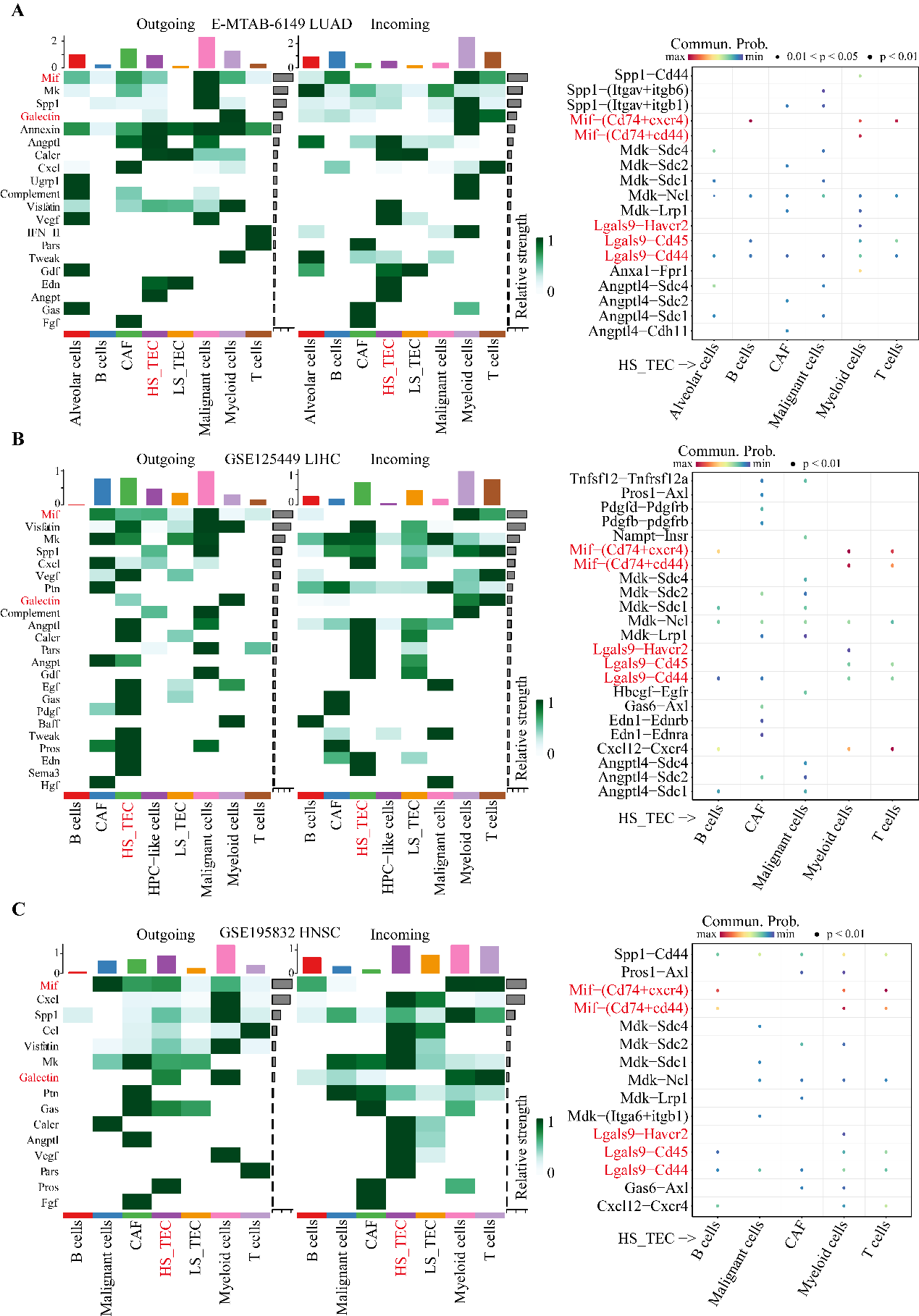


**Figure S2. Analysis of signaling pathways involved in cell-cell interactions.** Heatmaps show the outgoing (left) and incoming (right) signal strength of each signaling pathway among different cell types in lung cancer (A), liver cancer (B) and head and neck cancer (C). Bubble plots show all significant ligand-receptor pairs that contribute to the signaling sending from high-senescent tumor endothelial cells (HS-TEC) to other cell types in lung cancer (A), liver cancer (B) and head and neck cancer (C). The dot color and size in the bubble plot represent the communication probability and p-values, with blue and red corresponding to the minimum and maximum values, respectively.


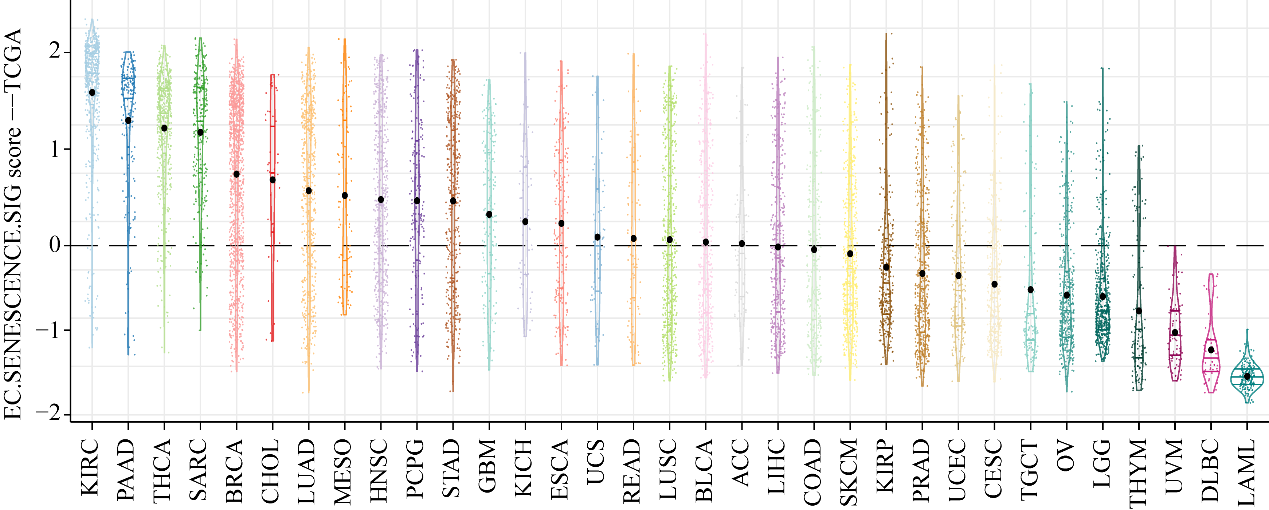


**Figure S3.** *EC.SENESCENCE.SIG* gsva score across 33 cancer types in TCGA pan-cancer cohorts.


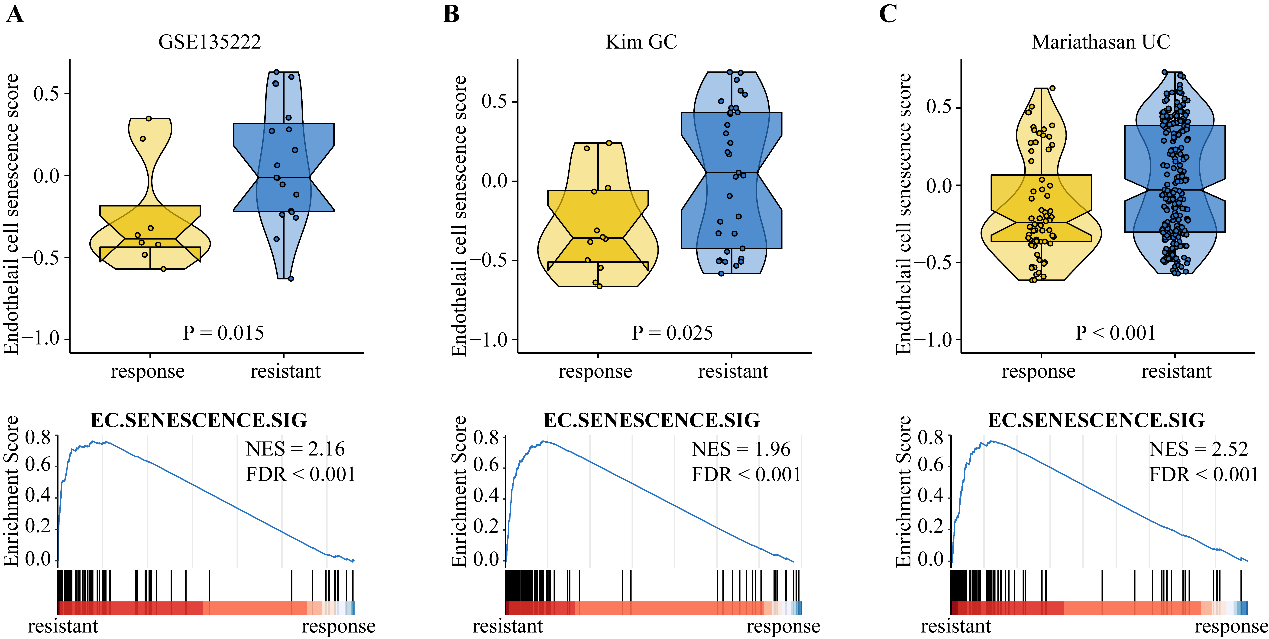


**Figure S4.** **Performance of the *EC.SENESCENCE.SIG*-dependent pan-cancer predictive model.** (A-C) The upper plots show the difference of *EC.SENESCENCE.SIG* scores in response and resistance to checkpoint immunotherapy groups. The differences were calculated by Wilcoxon rank sum test. The lower plots show the positive enrichment of *EC.SENESCENCE.SIG* in patients with resistance to checkpoint immunotherapy in lung cancer (GSE135222), gastric cancer (Kim GC) and urothelial carcinoma (Mariathasan UC) respectively. NES: Normalized enrichment score in the GSEA algorithm.


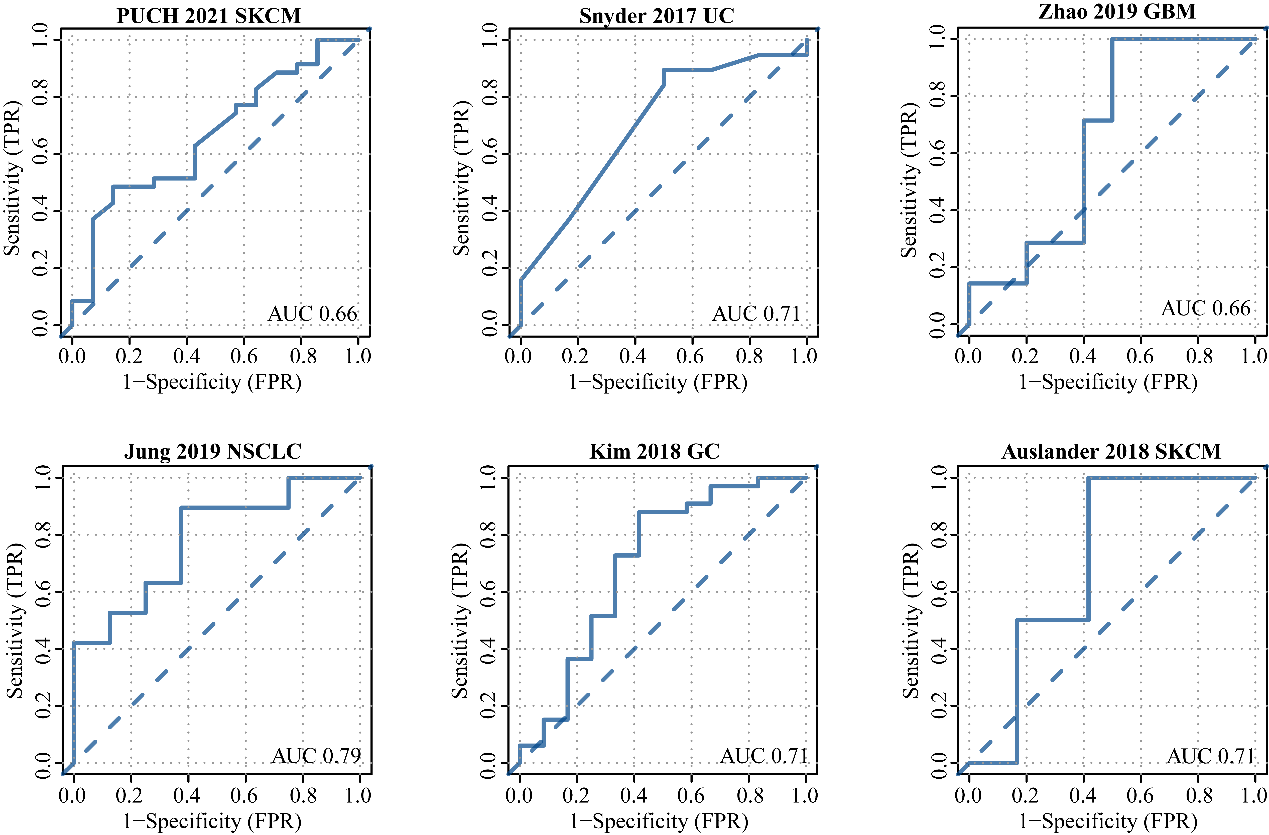


**Figure S5.** **Performance of the *EC.SENESCENCE.SIG*-dependent pan-cancer prognostic model.** Receiver operating characteristic (ROC) plots show the performance of the *EC.SENESCENCE.SIG* in distinguishing response and resistant to immunotherapy in six different cohorts. Area Under Curve (AUC) was calculated by ROC analysis and are displayed in the bottom right.


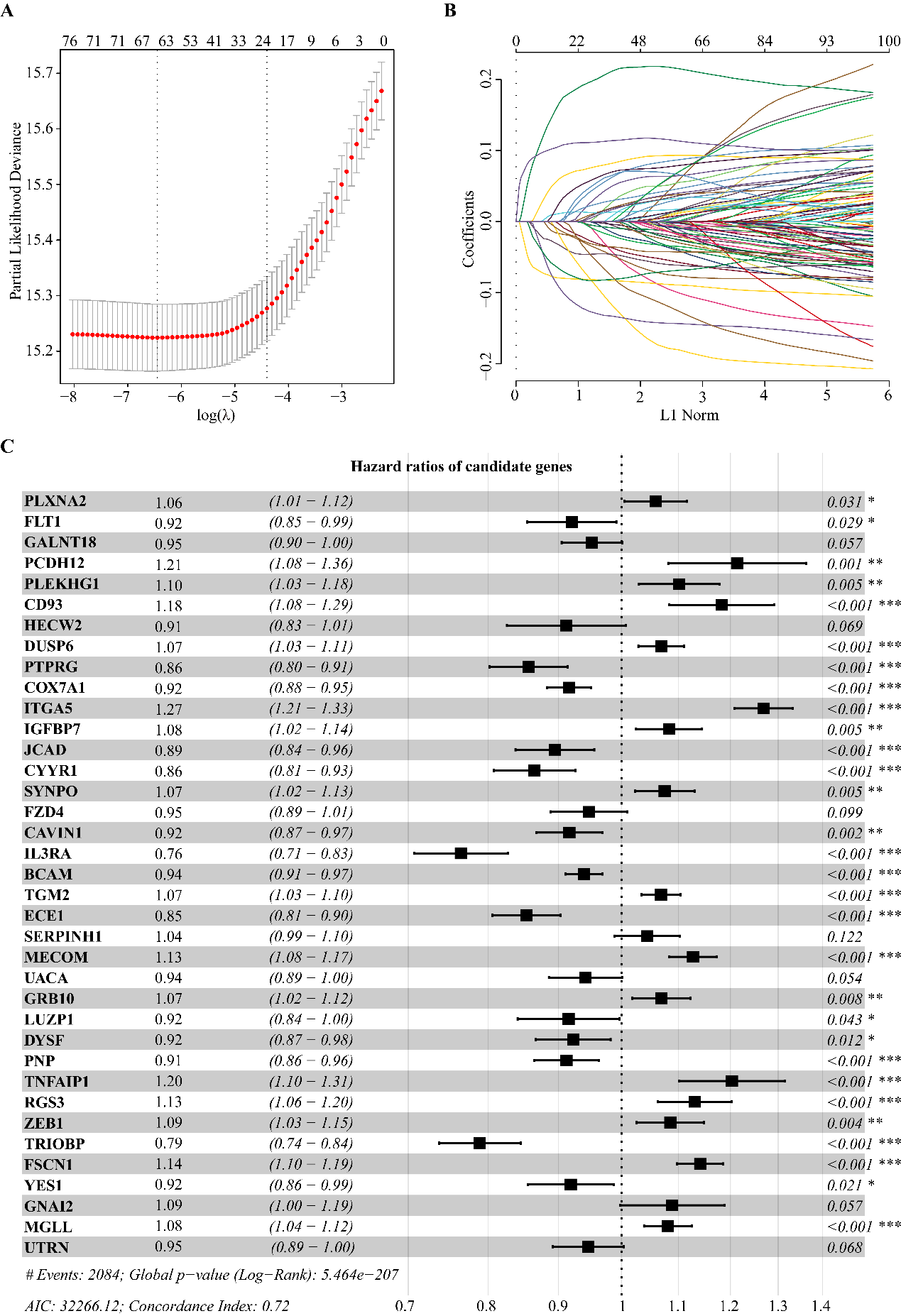


**Figure S6. Construction of a *EC.SENESCENCE.SIG*-related pan-cancer prognostic model** (**A-B**) LASSO coefficient profiles of the 50 selected genes in EC.SENESCENCE.SIG. 10-fold cross-validation to select tuning parameters for the LASSO model. (**C**) Forest plot of a multivariate Cox proportional hazards regression model in the overall survival of TCGA pan-cancer cohort.


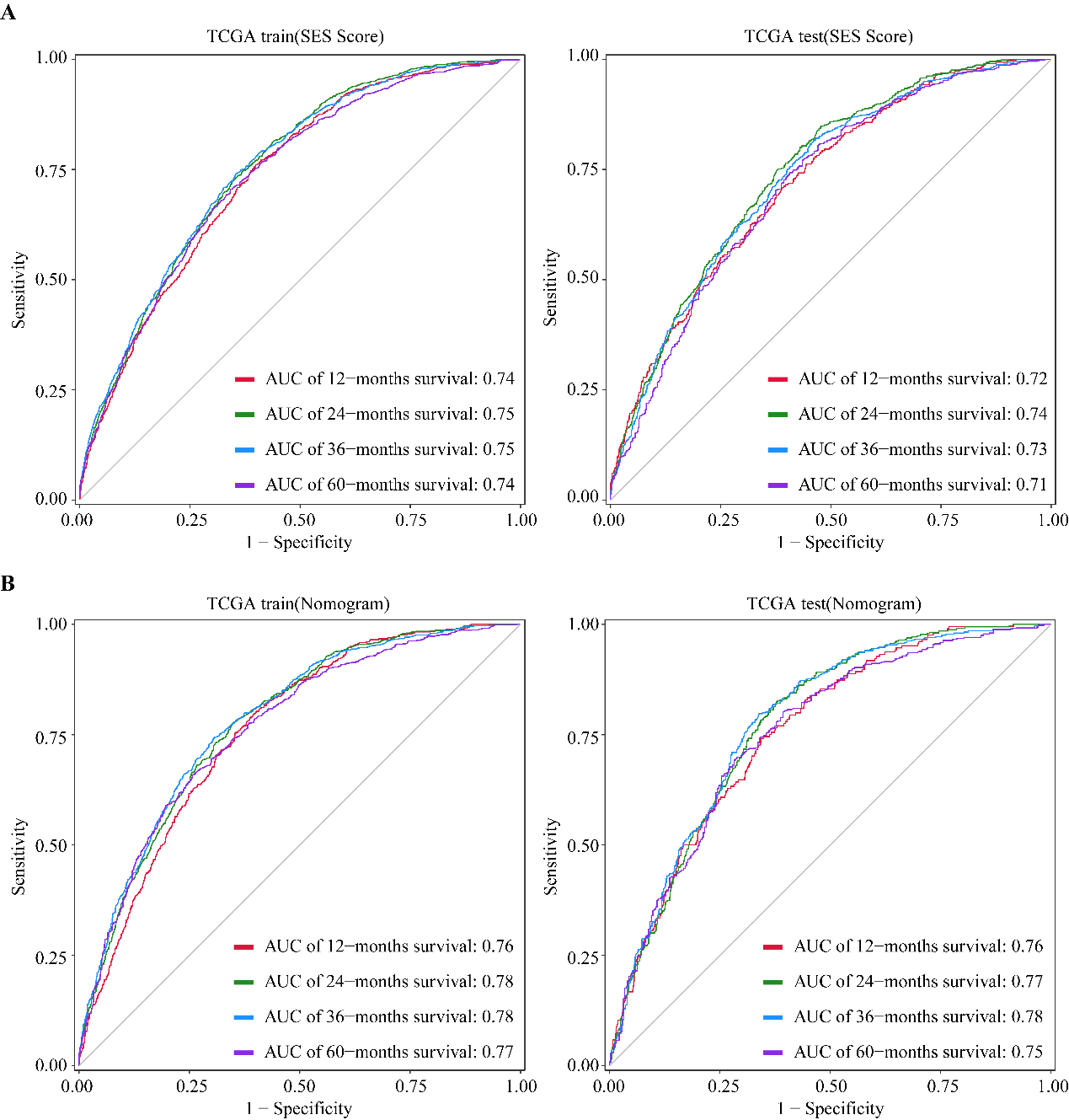


**Figure S7.** **Performance of the *EC.******SENESCENCE.SIG*-dependent pan-cancer prognostic model.** (A) Receiver operating characteristic (ROC) plots show the performance of the *EC.SENESCENCE.SIG*-related pan-cancer prognostic model in predicting overall survival of pan-cancer TCGA training and test cohorts. (B) ROC plots show the performance of the nomogram model in predicting overall survival of pan-cancer TCGA training and test cohorts. Area Under Curve (AUC) at 12 months, 24 months, 36 months and 60 months were calculated by ROC analysis and are displayed in the bottom right.


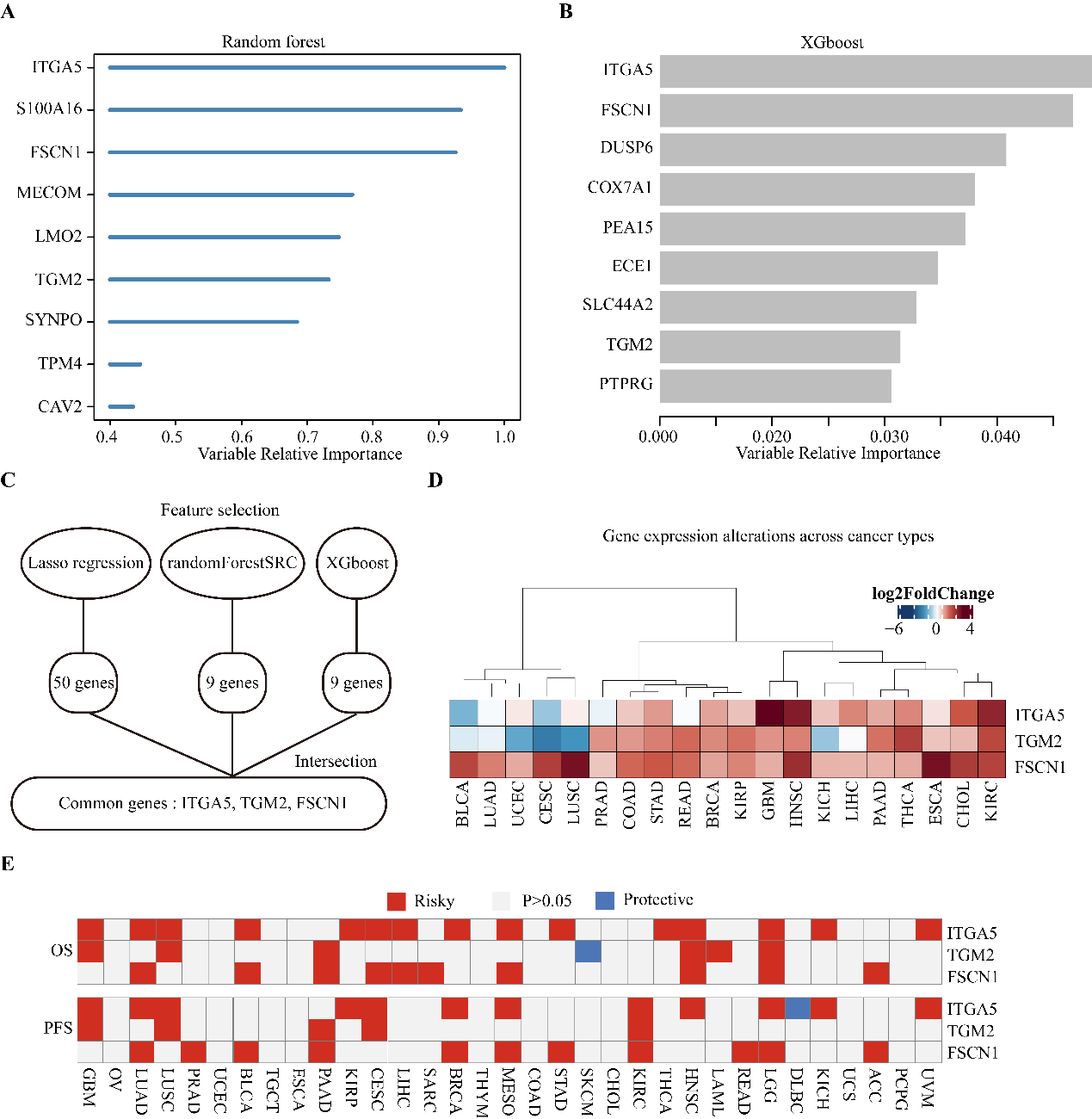


**Figure S8. Prognostic feature selection of *EC.SENESCENCE.SIG*.** Importance values ​​of selected genes in *EC.SENESCENCE.SIG* for patient prognosis assessment using random forest (**A**) or XGboost (**B**). (**C**) The flow chart shows the selection process of the three key genes in *EC.SENESCENCE.SIG* to predict OS of patients in the TCGA pan-cancer cohort. (**D**) Differential expression of these 3 genes in tumor tissue relative to normal tissue among 20 cancer types in the pan-cancer TCGA cohort. X-fold changes as compared to normal tissue are shown. (**E**) A summary of the relationship between expression of these 3 hub genes and patient prognosis (OS and PFS) across 33 cancer types in the TCGA pan-cancer cohort is shown.

**Additional References**

1. Hugo W, Zaretsky JM, Sun L, et al. Genomic and transcriptomic features of response to anti-PD-1 therapy in metastatic melanoma. *Cell* 2016;165(1):35-44.

2. Liu D, Schilling B, Liu D, et al. Integrative molecular and clinical modeling of clinical outcomes to PD1 blockade in patients with metastatic melanoma. *Nature medicine* 2019;25(12):1916-27.

3. Gide TN, Quek C, Menzies AM, et al. Distinct immune cell populations define response to anti-PD-1 monotherapy and anti-PD-1/anti-CTLA-4 combined therapy. *Cancer cell* 2019;35(2):238-55. e6.

4. Riaz N, Havel JJ, Makarov V, et al. Tumor and microenvironment evolution during immunotherapy with nivolumab. *Cell* 2017;171(4):934-49. e16.

5. Van Allen EM, Miao D, Schilling B, et al. Genomic correlates of response to CTLA-4 blockade in metastatic melanoma. *Science* 2015;350(6257):207-11.

6. Cui C, Xu C, Yang W, et al. Ratio of the interferon-γ signature to the immunosuppression signature predicts anti-PD-1 therapy response in melanoma. *NPJ genomic medicine* 2021;6(1):1-12.

7. Auslander N, Zhang G, Lee JS, et al. Robust prediction of response to immune checkpoint blockade therapy in metastatic melanoma. *Nature medicine* 2018;24(10):1545-49.

8. Mariathasan S, Turley SJ, Nickles D, et al. TGFβ attenuates tumour response to PD-L1 blockade by contributing to exclusion of T cells. *Nature* 2018;554(7693):544-48.

9. Snyder A, Nathanson T, Funt SA, et al. Contribution of systemic and somatic factors to clinical response and resistance to PD-L1 blockade in urothelial cancer: an exploratory multi-omic analysis. *PLoS medicine* 2017;14(5):e1002309.

10. Zhao J, Chen AX, Gartrell RD, et al. Immune and genomic correlates of response to anti-PD-1 immunotherapy in glioblastoma. *Nature medicine* 2019;25(3):462-69.

11. Kim ST, Cristescu R, Bass AJ, et al. Comprehensive molecular characterization of clinical responses to PD-1 inhibition in metastatic gastric cancer. *Nature medicine* 2018;24(9):1449-58.

12. Jung H, Kim HS, Kim JY, et al. DNA methylation loss promotes immune evasion of tumours with high mutation and copy number load. *Nature communications* 2019;10(1):1-12.

13. Braun DA, Hou Y, Bakouny Z, et al. Interplay of somatic alterations and immune infiltration modulates response to PD-1 blockade in advanced clear cell renal cell carcinoma. *Nature medicine* 2020;26(6):909-18.
